# Association of birthweight centiles and early childhood development of singleton infants born from 37 weeks of gestation in Scotland: A population-based cohort study

**DOI:** 10.1371/journal.pmed.1004108

**Published:** 2022-10-11

**Authors:** Abiodun Adanikin, Deborah A. Lawlor, Jill P. Pell, Scott M. Nelson, Gordon C. S. Smith, Stamatina Iliodromiti

**Affiliations:** 1 Centre for Healthcare Research, Institute of Health and Wellbeing, Coventry University, Coventry, United Kingdom; 2 Women’s Health Research Unit, Wolfson Institute of Population Health, Queen Mary University of London, London, United Kingdom; 3 MRC Integrative Epidemiology Unit at the University of Bristol, Bristol, United Kingdom; 4 Population Health Science, Bristol Medical School, University of Bristol, Bristol, United Kingdom; 5 Institute of Health and Wellbeing, University of Glasgow, Glasgow, United Kingdom; 6 School of Medicine, University of Glasgow, Glasgow, United Kingdom; 7 Department of Obstetrics and Gynaecology, University of Cambridge; NIHR Cambridge Biomedical Research Centre, Cambridge, United Kingdom; London School of Hygiene and Tropical Medicine, UNITED KINGDOM

## Abstract

**Background:**

Birthweight centiles beyond the traditional thresholds for small or large babies are associated with adverse perinatal outcomes but there is a paucity of data about the relationship between birthweight centiles and childhood development among children born from 37 weeks of gestation. This study aims to establish the association between birthweight centiles across the whole distribution and early childhood development among children born from 37 weeks of gestation.

**Methods and findings:**

This is a population-based cohort study of 686,284 singleton infants born from 37 weeks of gestation. The cohort was generated by linking pregnancy and delivery data from the Scottish Morbidity Records (2003 to 2015) and the child developmental assessment at age 2 to 3.5 years. The main outcomes were child’s fine motor, gross motor, communication, and social developmental concerns measured with the Ages and Stages Questionnaires—3 (ASQ-3) and Ages and Stages Questionnaire: Social & Emotional—2 (ASQ:SE-2), and for a subset of children with additional specialist tools such as the Modified Checklist for Autism in Toddlers (M-CHAT) if the ASQ3/SE indicate these are necessary. The ASQ score for each domain was categorised as “concern” and “no concern.”

We used multivariate cubic regression splines to model the associations between birthweight centiles and early childhood developmental concerns. We used multivariate Poisson regression models, with cluster robust errors, to estimate the relative risks (RRs) of developmental concerns below and above the established thresholds. We adjusted for maternal age, early pregnancy body mass index (BMI), parity, year of delivery, gestational age at delivery, smoking history, substance misuse in pregnancy, alcohol intake, ethnicity, residential area deprivation index, maternal clinical conditions in pregnancy (such as diabetes and pre-eclampsia), induction of labour, and child’s sex.

Babies born from 37 weeks of gestation with birthweight below the 25th centile, compared to those between the 25th and 74th centile, were at higher risk of developmental concerns. Those born between the 10th and 24th centile had an RR of 1.07 (95% CI: 1.03 to 1.12, *p* < 0.001), between the 3rd and 9th centile had an RR: 1.18 (95% CI: 1.12 to 1.25, *p* < 0.001), and <3rd centile had an RR of 1.37 (95% CI: 1.24 to 1.50, *p* < 0.001). There was no substantial increase in the risk of early childhood developmental concerns for larger birthweight categories of 75th to 89th (RR: 1.01; 95% CI: 0.97 to 1.05; *p* = 0.56), 90th to 96th (RR: 0.99; 95% CI: 0.94 to 1.05; *p* = 0.86), and ≥97th centiles (RR: 1.04; 95% CI: 0.97 to 1.12; *p* = 0.27), referent to birthweight between 25th and 74th centile.

The percentage of developmental concerns attributable to birthweight between the 10th and 24th centile was more than that of birthweight <3rd centile (*p* = 0.023) because this group includes more of the population. Approximately 2.50% (95% CI: 1.26 to 3.61) of social skills concerns and 3.00% (95% CI: 1.33 to 4.67) of fine motor developmental concerns were attributable to birthweight between the 10th and 24th centile compared to 0.90% (95% CI: 0.48 to 1.26) and 2.30% (95% CI: 1.73 to 2.67) respectively for birthweight <3rd centile. We acknowledge the limitation of ASQ as a screening tool, the subjective nature of developmental assessments (particularly for speech) among young children, and inability to control for early childhood illness and upbringing factors may have an impact on our findings.

**Conclusions:**

We observed that from 37 weeks of gestation birthweight below the 25th centile was associated with child developmental concerns, with an association apparent at higher centiles above the conventional threshold defining small for gestational age (SGA, 3rd or 10th centile). Mild to moderate SGA is an unrecognised potentially important contributor to the prevalence of developmental concerns. Closer surveillance, appropriate parental counselling, and increased support during childhood may reduce the risks associated with lower birthweight centiles.

## Abstract 2

In a population-based cohort study in Scotland, Abiodun Adanikin and colleagues study the association of birthweight centiles and motor, communication, and social developmental concerns identified at ages 2 to 3.5 years of age.

## Author summary

**Why was this study done?:**

- Existing studies investigating the association between birthweight centiles and childhood developmental concerns have focused mainly either on preterm babies or use established birthweight thresholds.
- We know little on how distribution of birthweights, across its entire range, associate with early childhood developmental concerns among infants born from 37 weeks of gestation.

**What did the researchers do and find?:**

- We analysed routinely collected maternity and delivery records of 686,284 singleton infants born from 37 weeks of gestation in Scotland between 2003 and 2015.
- Among these infants, we found that the association of birthweight with early childhood developmental concerns was apparent at higher centiles than the conventional threshold defining small for gestational age (<10th centile).
- More babies with birthweight between the 10th and 24th centile account for the burden of developmental concerns than those with birthweight <10th centile because this group includes more of the population.

**What do these findings mean?:**

- Although it is mostly unrecognised, mild to moderate small for gestational age (SGA) may be a key contributor to the burden of developmental concerns.
- Babies who had mild to moderate SGA may need closer monitoring and increased support in early childhood to reduce the risk of developmental concerns.

## Introduction

Low and high extremes of birthweight have been associated with adverse pregnancy and neonatal health outcomes, mortality, general wellbeing, and development [1]; however, there is sparse data regarding the shape and magnitude of the relationship between the distribution of birthweight centiles and subsequent childhood development. Available studies have mostly focused on prematurity [2,3] showing that preterm infants (<37 weeks’ gestational age) have delayed motor, social, and language development compared to their counterparts born at term (37 to 41 weeks) [4,5], and that low birthweight (LBW), defined as under 2,500 g (at any gestation) is a risk factor for developmental concerns [6]. A small number of studies have suggested that birthweight, even within the normal range, could be associated with adverse developmental and language outcomes [7,8]. However, these studies included both preterm and term births and did not explore how much of these associations were driven by gestational age or delivery events rather than birthweight centile.

Birthweights <10th percentile and >90th percentile or birthweight outside 2 standard deviations of the mean have mostly been used in clinical practice and research [1,9] for defining small and large for gestational age babies (SGA, LGA), respectively. Some studies, using these conventional thresholds for birthweight centiles, have examined the relationship between size at birth and neurodevelopment [10,11]. We have previously shown that thresholds used to define at risk birthweight should be derived from research that explores the whole birthweight distribution to ascertain group(s) at greatest risk and in relation to adverse perinatal outcomes found that the risk increases substantially below the 25th centile and above the 85th centile [12]. It is unknown whether the same associations are observed with subsequent childhood development. The aim of the study is to determine, in a large and contemporary population, how birthweight from 37 weeks of gestation, across its range, is associated with the main domains of childhood development at age 2 to 3.5 years. This could provide a means of early identification of infants at risk of developmental difficulties to enable early interventions and support that could ensure the child attains his or her optimal learning potential.

## Methods

### Study design and population

This study linked individual-level data from 5 Scotland-wide health databases: Scottish Morbidity Record 02 (SMR02), the Scottish Birth Record (SBR), the National Records for Scotland (NRS) death certificates, the Scottish Care Information Diabetes Collaboration (SCI-DC), and the Child Health Surveillance System Programme Pre-School (CHSP-PS), held by the Information Services Division of the National Health Service, Scotland. The Public Benefit and Privacy Panel for Health and Social Care of NHS Scotland gave approval to access and link the datasets for the purpose of the study and waived the requirement for individual consent (eDRIS_1617–0330). We did not request additional approval from an ethics committee. There was no prespecified analysis plan for the study. The study analyses were planned after data access and quality control checks, with input and agreement of the coauthors. The study is reported as per Strengthening the Reporting of Observational Studies in Epidemiology (STROBE) guidelines.

The study population was comprised of all singleton offspring who were delivered in Scottish maternity hospitals between 1 January 2003 and 31 December 2015 and had a child health assessment between the ages of 2 and 3.5 years. In total, 35 maternity hospitals were included, representing all possible maternity hospitals in Scotland at that time. The timing of child health assessment changed during the period of study, and child health datasets at 39 to 42 months (assessed approximately 1995 to 2007), 22 to 24 months (assessed approximately 1996 to 2007), 24 months (assessed approximately 2007 to 2014), and 27 to 30 months (assessed approximately 2013 onwards) were combined. The end date of deliveries for the birth cohort reflects the most recent data with available and complete child health follow-up at the time of data extraction.

All individuals registered with general practitioners in Scotland are allocated a unique identifier—the Community Health Index (CHI)—used for health care purposes and recorded in health databases. The CHI allows linkage of different databases at individual level using exact matching [13]. This enabled child health assessments to be linked with the maternity record in the SMR02 database via statutory birth registration information containing child and maternal CHI. The linked datasets provided comprehensive demographic and clinical information on births at Scottish maternity hospitals and subsequent child development. Further details on the datasets, the quality assurance procedures, and how to access data are available from the Public Health Scotland (www.publichealthscotland.scot).

We restricted the main analyses to singleton livebirths, delivered between and including the gestational ages of 37 weeks and 0 days (37^+0^) and 43 weeks and 6 days (43^+6^) during the study period. We considered that in practice, due to multiple factors including uncertainty about gestational age and maternal requests, few deliveries occur between 42^+0^ and 43^+6^. We excluded infants with known congenital anomaly, defined as infants with structural or genetic defects using ICD 10 codes Q00-Q99.

### Outcome

The primary outcomes of interest were the presence or absence of childhood developmental domains—*fine motor*, *gross motor*, *communication*, and *social skills*, captured in the child health databases from the health assessments carried out between the ages of 2 and 3.5 years. We did not consider the *problem-solving* domain as it was not captured in the child health surveys until recently. The universal child health reviews are a core element of the Child Health Programme in Scotland, and all assessments are routinely undertaken by trained health visitors using standardised methodology. Trained health visitors assessed childhood development in fine motor, gross motor, communication, and social skills domains. In assessing development, there is a structured discussion with parents to assess the extent to which children are attaining expected milestones and to elicit any concerns, then, the health visitor carefully observes/examines the children for presence of key skills, using developmental screening tool—the Ages and Stages Questionnaire (ASQ-3) [14–16]. When considered necessary, the health visitor supplements ASQ-3 with the Ages and Stages Questionnaire: Social & Emotional (ASQ:SE-2) or other more specialist tools such as the Modified Checklist for Autism in Toddlers (M-CHAT) [15–17]. The age of the child, corrected for gestational age at birth, was used for all assessments. At the end of a child health review, the health visitor uses the relevant Child Health Surveillance Programme–Preschool (CHSP-PS) review form to record their overall assessment of development in each domain as either “no concern” or “concern.” The categorisation of assessment result for each domain is determined by overall responses—including the opportunity to observe or examine for key skills, and the ASQ score cutoff for respective domain [18]. The childhood development data from CHSP-PS have been previously validated and has approximately 80% to 87% meaningful information recorded for all domains [19,20].

### Exposure

The main exposure was population birthweight centiles (standardised for sex and gestational age). Gestational age was defined in completed weeks based on earliest ultrasound estimation in the first half of pregnancy undertaken on more than 95% of women in the United Kingdom since the 1990s [21]. The centiles were calculated from distribution of birthweights in the study population. The population birthweight centile has been previously validated [12].

### Covariates

We considered that maternal age, early pregnancy body mass index (BMI), parity, year of delivery, gestational age at delivery, smoking history, substance misuse in pregnancy, alcohol intake, ethnicity, residential area deprivation index, diabetes in pregnancy, pre-eclampsia, maternal infections during pregnancy (including viral hepatitis, HIV/AIDS and other sexually transmitted infections (STIs), and tuberculosis), history of previous stillbirth or spontaneous miscarriage, induction of labour, and child’s sex could confound the relationship between birthweight and child developmental outcomes, on the basis that they plausibly influence birthweight, and child motor and cognitive development. Maternal diabetic status was ascertained from ICD 10 codes (O244, O249, E10-14, O240-1, O243) recorded in the SMR02 database and the SCI-DC diabetes register, as were pre-eclampsia/eclampsia (O140-2, O149, O150-1, O11, O11X) and maternal infections (O980-9). The Scottish Index of Multiple Deprivation (SIMD) was used as a measure of socioeconomic status. The SIMD of an area of residence is derived from data across 7 domains: income, employment, education, health, access to services, crime, and housing and was categorised into tenths of the distribution, with the lowest decile representing that the woman lives in one of the most deprived areas of Scotland [22]. Ethnicity was recoded as “white,” “black,” “Asian,” “mixed and other,” as defined by the Scottish Government for census purposes [23]. Ethnicity has previously been shown to be independently associated with adverse perinatal outcomes [24]. We considered ethnicity since it could influence birthweight and childhood development (e.g., through language barriers and access to services).

All variables were examined for data entry errors, guided by medical and biological plausibility. Where possible, related variables were used to cross check any entries that were outside the plausible range and if these were deemed to be valid, they were retained. The remaining questionable data were converted to “missing” (S1 Table provides details of the plausible ranges/categorisation for all variables).

### Statistical analyses

We used multivariate cubic regression splines to model the associations between birthweight centiles and each child developmental outcome, using the *mvrs* command in Stata. The *mvrs* selects the regression spline model that best predicts the outcome variable from one or more independent variables, at least one of which should be continuous. The programs use basis functions for regression splines to automatically determine the position of knots for the equally spaced birthweight centiles [25]. In these analyses, we selected the position of the knots in the spline model as being where the association between birthweight centile and child developmental outcomes changed in direction or magnitude. With the identification of the knots, we used multivariate Poisson regression models to estimate the relative risks of developmental concerns below and above the chosen knots, referent to birthweight centiles between the thresholds. We used cluster robust errors in these models to account for the fact that we were analysing data for children and some women will have had more than one child over the study period. We anticipated that the cubic spline modelling might not identify knots at the thresholds used currently in clinical practice to define SGA (<10th centile and <3rd centile) and LGA age (>90th centile and ≥97th) babies. Therefore, a priori we decided to estimate the relative risks of developmental concerns using birthweight centile categories of <3rd, ≥3rd to <10th, 90th to 96th and ≥97th and compare with the results driven by the cubic spline knots.

We estimated the population attributable fraction (PAF) of developmental concerns for the birthweight categories. PAF is the estimated fraction of all cases that would not have occurred if the infants in the birthweight centile of interest (exposure) had a birthweight between 25th and 74th centile (referent category), calculated as PAF = p_c_(1-1/RR), where p_c_ is the prevalence of exposure among cases (i.e., children with developmental concerns), and RR is the adjusted relative risk for the birthweight centile of interest [26,27]. All analyses were performed using Stata (version 16, StataCorp LP, College Station, Texas, United States of America). The level of statistical significance was set at 0.05, using a 2-sided alternative hypothesis.

### Supplementary analyses

Mode of delivery, use of analgesia/anaesthesia in labour, Apgar score <7 at 5 minutes, and neonatal unit (NNU) admission have previously been associated with childhood development [28,29]. Since birthweight could influence the choice of delivery method, use of analgesia/anaesthesia in labour, Apgar score, and neonatal admission, which in turn could account for variation in the dependent variable (i.e., developmental outcomes), we explored the potential mediation by including these variables in the model after adjusting for confounders [30]. Mode of delivery was a categorical variable, recorded as “spontaneous cephalic,” “instrumental cephalic,” “assisted vaginal breech,” “elective cesarean section,” and “emergency cesarean section” deliveries. Analgesia/anaesthesia in labour was recorded as “none,” “opiates,” “gas and air only,” “spinal (including combined anaesthesia),” and “general anaesthesia.” Apgar score <7 at 5 minutes and NNU admission were coded as “No” and “Yes.”

To explore whether the magnitude of the associations was different among the whole birth population (inclusive of preterm births), we performed supplementary analysis for gestational ages 28 to 43 weeks. Based on peer reviewers’ comments, we conducted additional analyses restricting analyses to babies born between 37^+0^ and 41^+6^ weeks, controlling for child’s age at developmental assessment, assessing the relationship between absolute birthweight in kilograms and developmental concerns (using categories <2.5 kg and >4.0 kg, referent to 2.5 to 4.0 kg, and categories <2.5 kg, between 2.5 and 3.0 kg and >4.0 kg, referent to 3.0 to 4.0 kg), and checking for any differences in associations with developmental concerns within the 25th to 74th percentile range (using the categories 25th to 34th, 35th to 44th, 55th to 64th, 65th to 74th, referent to 45th to 54th birthweight centile). These additional analyses were considered to examine the associations between birthweight centiles and early childhood development in a typical term population (37^+0^ and 41^+6^ weeks), whether the timing of developmental assessments influence the observed associations, whether the associations differ using absolute birthweight compared to using birthweight centiles, and if there were differences in the association of birthweight centiles with various outcome domains within the referent group (25th to 74th centile).

### Missing data

The extent of missingness among variables varied, ranging from 0% (maternal age, gestational age, child sex, and birthweight) to 57% (for outcome variables). We examined differences in distributions of variables between children with and without missing outcomes and whether the missingness of outcome variables is predicted by child’s birthweight, gestational age at birth, or year of birth. Multiple imputation by chained equations (MICE) was used to predict missing data for covariates and outcomes with missing >0% [31]. All variables included in any of the analysis models, including birthweight and the outcomes, were used to predict the distribution of each missing value. We generated 20 imputed datasets with 10 iterations for each imputation [32]. We present results for the imputed datasets (MI) involving all babies born from 37 weeks of gestation (*N* = 686,284) in supplementary analyses. MI analyses assume that data are missing at random (i.e., that missingness is only influenced by observed variables included in our imputation model) [33]. On the other hand, complete case analyses assumes that missingness would not influence an outcome conditional on the main exposure and co-variables that are adjusted for in each analysis [33].

## Results

A total of 759,641 deliveries occurred between 1 January 2003 and 31 December 2015. After applying prespecified exclusions, there were 686,284 singleton infants born from 37 weeks of gestation and 40,717 singleton preterm births (<37 weeks of gestation). Of the infants born from 37 weeks of gestation, 14,571 (2.12%) had birthweight <2.5 kg, 576,019 (83.93%) between 2.5 and 4.0 kg, and 95,694 (13.94%) with birthweight >4.0 kg. The distribution of birthweight percentiles of the infants and corresponding birthweight in grams is summarised in S2 Table.

**Fig 1:**
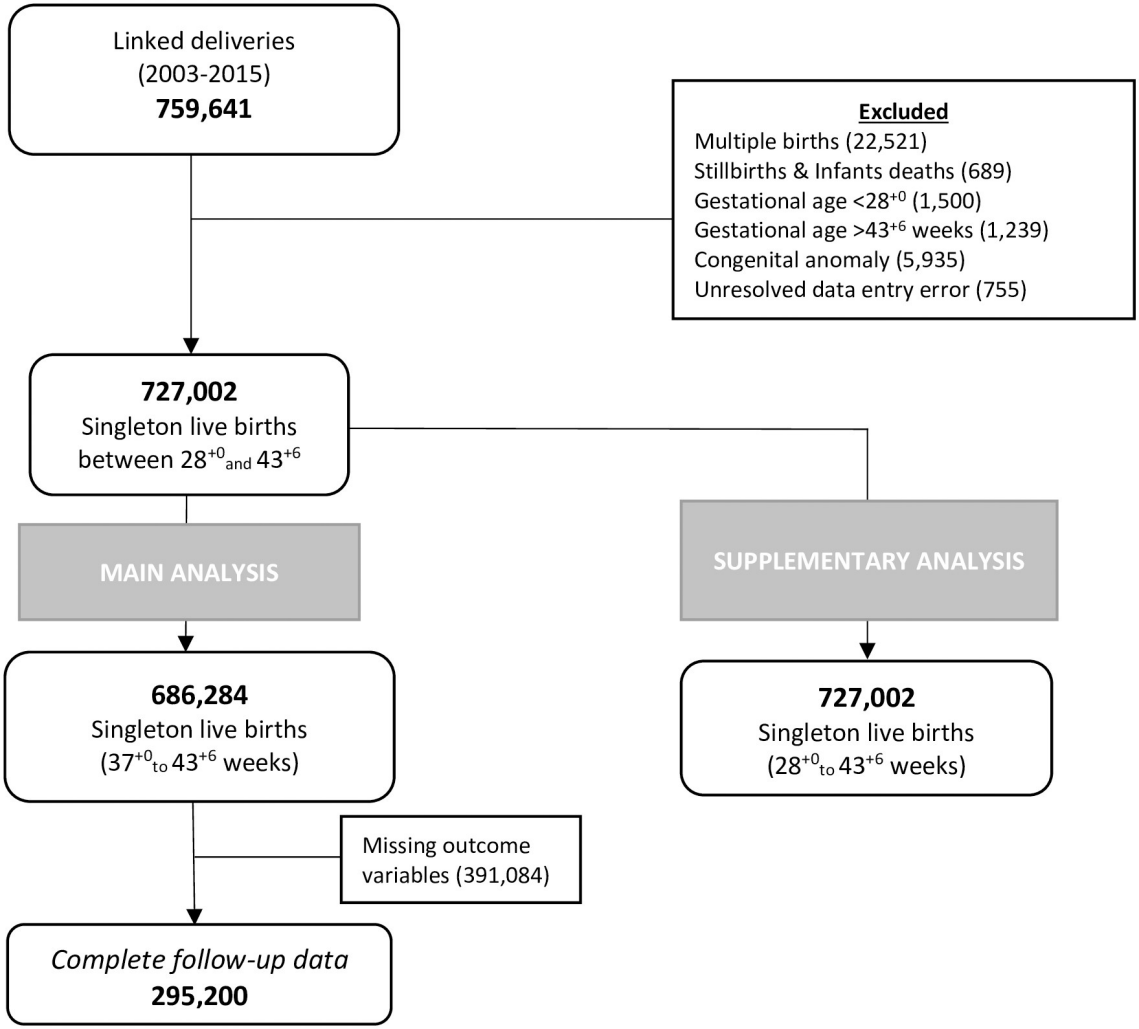
Eligible cohort and analysis sample flow chart.

A total of 295,200 (43.00%) of those born from 37 weeks of gestation completed child developmental assessments at age 2 to 3.5 years (Fig 1). Of these, 93,873 (31.80%) had developmental assessment at approximately 22 to 24 months, 180,700 (61.21%) at 27 to 30 months and 20,627 (6.99%) at 39 to 42 months. A total of 41,877 (14.19%) had concerns recorded in at least 1 domain: 7,033 (2.38%) had fine motor concern, 5,957 (2.02%) gross motor concern, 36,550 (12.38%) communication concern, and 10,865 (3.68%) social skills concern.

A summary of variable missingness is shown in S3 Table, and the distributions of variables among children with and without missing outcomes are presented in S4 Table. Births in the earliest years of our analysis cohort (2003 to 2007) were more likely to have missing outcomes (S5 Table).

The characteristics of the study population by developmental concern are presented in Table 1. Early childhood developmental concerns were commoner among boys, children delivered following induction of labour with Apgar score <7 at 5 minutes, admitted to NNU, who had multiparous mothers, with a maternal history of smoking or substance misuse during pregnancy, and who lived in more deprived conditions.

**Table 1:** Basic maternal and child characteristics of infants with complete follow-up data by developmental concern.

Variable	Frequency, n	Developmental concern	
		*No*	*Yes*
**Maternal age** (years)	295,200	28.94 ± 6.02	27.88 ± 6.13
**Maternal BMI** (kg/m^2^)	225,358	26.05 ± 5.58	26.78 ± 6.15
**Parity**	292,968		
Nulliparous		113,746 (45.26)	16,625 (39.94)
Multiparous		137,592 (54.74)	25,005 (60.06)
**Year of birth**	295,200		
2003–2007		64,918 (25.63)	9,749 (23.28)
2008–2011		67,336 (26.58)	12,087 (28.86)
2012–2015		121,069 (47.79)	20,041 (47.86)
**Gestational age** (weeks)	295,200		
37^+0^ to 38^+6^		45,478 (17.95)	9,071 (21.66)
39^+0^ to 40^+6^		141,805 (55.98)	23,151 (55.28)
41^+0^ to 41^+6^		59,226 (23.38)	8,683 (20.73)
42^+0^ to 43^+6^		6,814 (2.69)	972 (2.32)
**Smoking history**	281,761		
Never smoked		160,107 (66.16)	22,570 (56.75)
Former smoker		28,110 (11.62)	4,453 (11.2)
Current smoker		53,772 (22.22)	12,749 (32.06)
**Substance misuse in pregnancy**	191,622		
No		161,008 (97.86)	26,061 (96.21)
Yes		3,526 (2.14)	1,027 (3.79)
**Weekly alcohol intake**	190,185		
None		153,411 (93.85)	25,308 (94.72)
1–2 units		3,248 (1.99)	407 (1.52)
≥3 units		6,808 (4.16)	1,003 (3.75)
**SIMD Decile**	294,839		
1		36,496 (14.42)	8,587 (20.53)
2		32,056 (12.67)	6,647 (15.89)
3		27,608 (10.91)	5,475 (13.09)
4		25,615 (10.12)	4,534 (10.84)
5		24,329 (9.62)	3,959 (9.47)
6		22,012 (8.7)	3,132 (7.49)
7		22,137 (8.75)	2,964 (7.09)
8		21,830 (8.63)	2,572 (6.15)
9		21,543 (8.51)	2,204 (5.27)
10		19,389 (7.66)	1,750 (4.18)
**Induction of labour**	293,370		
No		184,742 (73.39)	30,114 (72.29)
Yes		66,969 (26.61)	11,545 (27.71)
**Child’s sex**	295,200		
Male		122,331 (48.29)	28,513 (68.09)
Female		130,992 (51.71)	13,364 (31.91)
**Apgar score (5 minutes)**	292,949		
7–10		248,757 (98.93)	40,814 (98.35)
<7		2,692 (1.07)	686 (1.65)
**Birthweight centiles**	295,200		
<3rd		4,268 (1.68)	1,131 (2.70)
3rd–9th		16,171 (6.38)	3,346 (7.99)
10th–24th		36,550 (14.43)	6,686 (15.97)
25th–74th		127,635 (50.38)	20,154 (48.13)
75th–89th		39,263 (15.50)	6,052 (14.45)
90th–96th		18,539 (7.32)	2,798 (6.68)
≥97th		10,897 (4.30)	1,710 (4.08)
**NNU admission**	290,682		
Not admitted		237,117 (95.05)	38,206 (92.81)
Admitted (up to 48 hours)		7,178 (2.88)	1,409 (3.42)
Admitted (beyond 48 hours)		5,169 (2.07)	1,549 (3.76)

n (%)–percentage presented in columns.

BMI, body mass index; NNU, neonatal unit; SIMD, Scottish Index of Multiple Deprivation.

**Fig 2:**
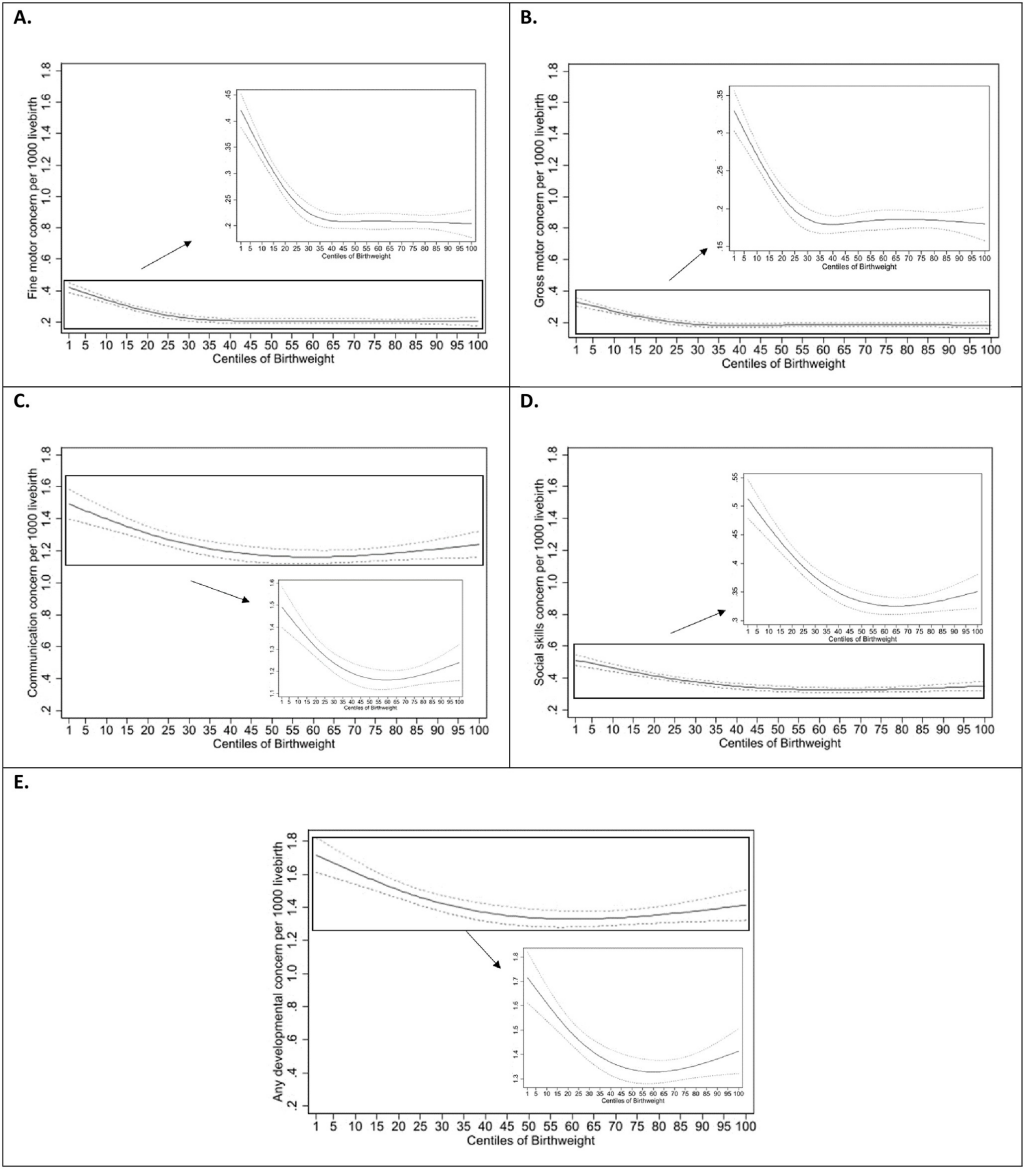
Regression splines showing child developmental concerns relative to birthweight centiles. The bold lines represent the predicted occurrence of developmental concerns, and the dotted lines represent the 95% confidence interval. **(A)** Restricted cubic spline showing predicted relationship between birthweight centiles and fine motor concern. **(B)** Restricted cubic spline showing predicted relationship between birthweight centiles and gross motor concern. **(C)** Restricted cubic spline showing predicted relationship between birthweight centiles and communication concern. **(D)** Restricted cubic spline showing predicted relationship between birthweight centiles and social skills concern. **(E)** Restricted cubic spline showing predicted relationship between birthweight centiles and any developmental concern.

Fig 2 shows the shape of the relationships between birthweight and the developmental outcomes.

Reverse J shaped curves were observed for fine and gross motor developmental concerns and hockey stick shape for communication and social skills developmental concerns. On visual inspection, there was a steep negative linear association up to 25th centile for all outcomes and a less steep positive linear association above 75% centile for communication and social skills developmental concerns.

The proportion of children by birthweight centiles with developmental concern in each domain is summarised in S6 Table. Table 2 shows higher relative risk of developmental concerns for birthweight categories below the 25th centile. Children with birthweight <3rd centile had approximately 40% higher risk of developmental concern (RR: 1.37; 95% CI: 1.28 to 1.47; *p* < 0.01) referent to birthweight between the 25th and 74th centiles, for complete case analysis. In particular, their risk of fine and gross motor concerns was double the risk among children in the referent birthweight category (25th to 74th centile). Those with birthweights between the 3rd and 9th centiles had more than 40% risk of fine motor (RR: 1.43; 95% CI: 1.24 to 1.65; *p* < 0.01) and gross motor (RR: 1.41; 95% CI: 1.21 to 1.64, *p* < 0.01) concerns, and 30% (RR: 1.31; 95% CI: 1.18 to 1.46; *p* < 0.01) risk of social skills concern, while children with birthweights between the 10th and 24th centiles had 21% (RR: 1.21; 95% CI: 1.08 to 1.35; *p* < 0.01) higher risk of fine motor concerns compared to the referent category (25th to 74th centile). There was no substantial increase in the risk of early childhood development concerns for larger birthweight categories of 75th to 89th (RR: 1.01; 95% CI: 0.97 to 1.05; *p* = 0.56), 90th to 96th (RR: 0.99; 95% CI: 0.94 to 1.05; *p* = 0.86), and ≥97th centiles (RR: 1.04; 95% CI: 0.97 to 1.12; *p* = 0.27) after adjustment for confounders, compared to the referent group (25th to 74th centile). The regression results for the imputed datasets were directionally similar with the complete case analyses (S7 Table).

**Table 2:** Adjusted RR with 95% confidence intervals of developmental concerns at chosen birthweight centiles referent to the birthweight between 25th and 74th centiles (for gestational age 37^+0^ to 43^+6^).

	Birthweight centile	Risk of any developmental concern		Risk for each domain							
				Fine motor concern		Gross motor concern		Communication concern		Social skills concern	
		*RR (95% CI)*	*p value*	*RR (95% CI)*	*p value*	*RR (95% CI)*	*p value*	*RR (95% CI)*	*p value*	*RR (95% CI)*	*p value*
**Unadjusted analysis** §	25th–74th (ref)										
	<3rd	1.54 (1.46–1.62)	<0.001	2.68 (2.39–3.00)	<0.001	2.40 (2.11–2.73)	<0.001	1.52 (1.43–1.61)	<0.001	1.79 (1.61–2.00)	<0.001
	3rd–9th	1.26 (1.22–1.30)	<0.001	1.64 (1.51–1.78)	<0.001	1.52 (1.39–1.66)	<0.001	1.26 (1.22–1.31)	<0.001	1.46 (1.36–1.56)	<0.001
	10th–24th	1.13 (1.11–1.16)	<0.001	1.36 (1.28–1.45)	<0.001	1.30 (1.21–1.39)	<0.001	1.13 (1.09–1.16)	<0.001	1.25 (1.19–1.32)	<0.001
	75th–89th	0.98 (0.95–1.01)	0.128	0.93 (0.86–1.00)	0.044	0.98 (0.91–1.06)	0.614	0.99 (0.96–1.02)	0.355	0.94 (0.89–1.00)	0.047
	90th–96th	0.96 (0.93–1.00)	0.038	0.88 (0.79–0.98)	0.015	0.93 (0.83–1.04)	0.194	0.96 (0.93–1.00)	0.064	0.93 (0.86–1.00)	0.066
	≥97th	0.99 (0.95–1.04)	0.820	1.00 (0.89–1.13)	0.974	0.98 (0.85–1.12)	0.729	1.01 (0.96–1.06)	0.837	0.97 (0.88–1.07)	0.576
**Adjusted analysis** ¥	25th–74th (ref)										
	<3rd	1.37 (1.24–1.50)	<0.001	2.05 (1.65–2.54)	<0.001	2.33 (1.87–2.90)	<0.001	1.34 (1.21–1.49)	<0.001	1.43 (1.19–1.72)	<0.001
	3rd–9th	1.18 (1.12–1.25)	<0.001	1.43 (1.24–1.65)	<0.001	1.41 (1.21–1.64)	<0.001	1.18 (1.12–1.26)	<0.001	1.31 (1.18–1.46)	<0.001
	10th–24th	1.07 (1.03–1.12)	0.001	1.21 (1.08–1.35)	0.001	1.16 (1.03–1.31)	0.012	1.07 (1.02–1.11)	0.005	1.17 (1.08–1.27)	<0.001
	75th–89th	1.01 (0.97–1.05)	0.557	1.05 (0.94–1.18)	0.364	1.02 (0.90–1.15)	0.777	1.02 (0.97–1.06)	0.497	1.08 (0.99–1.17)	0.071
	90th–96th	0.99 (0.94–1.05)	0.862	1.00 (0.85–1.17)	0.992	0.90 (0.76–1.07)	0.220	1.00 (0.94–1.06)	0.961	1.04 (0.93–1.17)	0.505
	≥97th	1.04 (0.97–1.12)	0.271	1.06 (0.88–1.29)	0.533	1.13 (0.93–1.37)	0.208	1.05 (0.97–1.13)	0.204	1.07 (0.92–1.24)	0.390

^§^ Unadjusted, CCA, *n* = 295,200.

^¥^ Adjusted CCA, *n* = 118,325.

Analysis was adjusted for maternal age, BMI, parity, year of birth, gestational age at delivery, child’s sex, smoking, substance misuse in pregnancy, alcohol intake, socioeconomic status, ethnicity, diabetes, pre-eclampsia, maternal infection during pregnancy, history of stillbirth and spontaneous abortion, and induction of labour.

BMI, body mass index; CCA, complete case analysis; RR, relative risk.

When potential mediators—mode of delivery, use of analgesia/anaesthesia in labour, Apgar score at 5 minutes and NNU admission—were included in the regression model, there was a marginal reduction in the magnitude of the associations between birthweight centiles and developmental concerns (S8 Table). Adjustment for the child’s age at developmental assessment did not alter the direction and magnitude of associations (S8 Table).

In additional analyses using thresholds of birthweight to define low birthweight (<2.5 kg) and high birthweight (>4.0 kg), referent to birthweight between 2.5 and 4.0 kg, we found babies with birthweight <2.5 kg were at increased risk of developmental concerns (S9 Table). With more granular categories of birthweight using <2.5 kg, between 2.5 to 3.0 kg (approximately corresponding to the population birthweight between the 10th and 24th centiles), and >4.0 kg, referent to birthweight between 3.0 and 4.0 kg, there was similarity in direction of associations of birthweight <2.5 kg and between 2.5 and 3.0 kg with developmental concerns. However, the magnitude of associations was higher among children with birthweight <2.5 kg (S10 Table). After adjusting for confounders, babies with birthweight between 2.5 and 3.0 kg had 12% higher risk of developmental concerns (RR: 1.12; 95% CI: 1.08 to 1.17; *p* < 0.01) than the referent category of birthweight between 3.0 and 4.0 kg.

The proportion of children with developmental concerns and regression for babies born between the gestational ages of 37^+0^ to 41^+6^ is presented in S11 Table. The result of regression analysis for these babies were similar to our main analysis using gestational ages 37^+0^ to 43^+6^ (S12 Table). In supplementary analysis involving all births (inclusive of preterm births), the results of complete case analysis and imputed datasets were consistent with analyses involving pregnancies from 37 weeks of gestation only (S13 Table). There were no differences in associations with developmental concerns within the referent group, 25th to 74th percentile (S14 Table). The distribution of birthweights between the 25th and 74th centiles was not associated with developmental concerns.

The percentage of developmental concerns attributable to birthweight between the 10th and 24th centile was more than that of birthweight <3rd centile (*p* = 0.023). Approximately 0.80% (95% CI: 0.58 to 1.00) of developmental concerns in babies born from 37 weeks of gestation were attributable to birthweight <3rd centile while 1.05% (95% CI: 0.47 to 1.71) were attributable to birthweight between 10th and 24th centile, in adjusted analysis (Table 3). The burden of communication concerns (*p* = 0.010) and social skills concerns (*p* < 0.001) were similarly higher for birthweight between 10th and 24th centile than for birthweight <3rd centile. There was no difference in the PAF of fine and gross motor concerns. At the other end of the birthweight range, the burden of developmental concerns associated with birthweight ≥75th centile was less than 1%. In supplementary analysis involving all births (inclusive of preterm babies), 2.94% (1.43 to 4.16) of fine motor concerns, 1.73% (0.00 to 3.10) of gross motor concerns, and 1.53% (0.45 to 2.57) of social skills concerns were attributable to birthweight between 10th and 24th centile in babies born from 37 weeks of gestation, in adjusted analysis (S15 Table).

**Table 3:** PAFs of developmental concerns.

	Birthweight centile	PAF for any developmental concern	PAF for each domain			
			*Fine motor concern*	*Gross motor concern*	*Communication concern*	*Social skills concern*
**Unadjusted analysis**	<3rd	1.05 (0.95 to 1.15)	2.76 (2.56 to 2.93)	2.33 (2.1 to 2.53)	1.03 (0.90 to 1.14)	1.32 (1.14 to 1.5)
	3rd–9th	1.65 (1.44 to 1.85)	3.90 (3.38 to 4.38)	3.08 (2.53 to 3.58)	1.65 (1.44 to 1.89)	2.84 (2.38 to 3.23)
	10th–24th	1.84 (1.59 to 2.21)	4.76 (3.94 to 5.59)	3.92 (2.95 to 4.77)	1.84 (1.32 to 2.21)	3.40 (2.71 to 4.12)
	75th–89th	−0.31 (−0.79 to 0.15)	−0.98 (−2.12 to 0.00)	−0.29 (−1.38 to 0.79)	−0.15 (−0.63 to 0.29)	−0.89 (−1.73 to 0.00)
	90th–96th	−0.29 (−0.53 to 0.00)	-0.82 (−1.59 to −0.12)	−0.45 (−1.23 to 0.23)	−0.29 (−0.53 to 0.00)	−0.45 (−0.98 to 0.00)
	≥97th	−0.04 (−0.21 to 0.15)	0.00 (−0.49 to 0.46)	−0.08 (−0.71 to 0.43)	0.04 (−0.17 to 0.23)	−0.12 (−0.55 to 0.26)
**Adjusted analysis** ¥	<3rd	0.81 (0.58 to 1.00)	2.25 (1.73 to 2.67)	2.28 (1.86 to 2.62)	0.76 (0.52 to 0.99)	0.90 (0.48 to 1.26)
	3rd–9th	1.22 (0.86 to 1.60)	3.01 (1.94 to 3.94)	2.62 (1.56 to 3.51)	1.22 (0.86 to 1.65)	2.13 (1.37 to 2.84)
	10th–24th	1.05 (0.47 to 1.71)	3.12 (1.33 to 4.67)	2.34 (0.50 to 4.02)	1.05 (0.31 to 1.59)	2.47 (1.26 to 3.61)
	75th–89th	0.15 (−0.46 to 0.71)	0.62 (−0.83 to 1.98)	0.27 (−1.56 to 1.83)	0.29 (−0.46 to 0.85)	1.04 (−0.14 to 2.03)
	90th–96th	−0.07 (−0.45 to 0.33)	0.00 (−1.06 to 0.87)	−0.67 (−1.89 to 0.39)	0.00 (−0.45 to 0.4)	0.23 (−0.45 to 0.87)
	≥97th	0.15 (−0.12 to 0.43)	0.23 (−0.55 to 0.90)	0.46 (−0.30 to 1.08)	0.19 (−0.12 to 0.46)	0.26 (−0.35 to 0.77)

^¥^ Adjustment for maternal age, BMI, parity, year of birth, gestational age at delivery, child’s sex, smoking, substance misuse in pregnancy, alcohol intake, socioeconomic status [deprivation index], ethnicity, diabetes, pre-eclampsia, maternal infection during pregnancy, history of stillbirth and spontaneous abortion, and induction of labour, referent to birthweight between 25th and 74th centile.

Result presented as percent (95% CI).

BMI, body mass index; PAF, population attributable fraction.

## Discussion

We found that while the risk of developmental concerns was increased at birthweight less than 25th centile threshold compared to those between 25th and 74th centile thresholds, the risk is not dichotomous but rather a continuum, with the magnitude increasing progressively as birthweight centile falls below this threshold. Therefore, from individual child’s perspective, the distance they fall below this threshold is important with respect to developmental monitoring. Although the risk of developmental concerns increases as birthweight falls, the percentage of developmental concerns attributable to birthweights between the 10th and 24th centile in infants born from 37 weeks of gestation is higher because this group includes more of the population. Hence, from a population perspective preventing mild to moderate SGA from 37 weeks of gestation or reducing its effect would make a bigger impact than targeting only extremes of birthweight.

The commonest method of summarising birthweight categories is by using thresholds of absolute weight, usually <2,500 grams for low birthweight and >4,000 grams for high birthweight, or percentile, usually <10th or <3rd centile for SGA, and >90th or >97th centile for LGA, which oversimplifies the relationship between birthweights and health or developmental outcomes. As shown in this study and elsewhere [12,34], a nonlinear relationship exists between birthweight centiles as a continuous measure and short- to long-term birth outcomes. Hence, in certain situations when birthweight centiles are dichotomised, critical information about how birthweight centiles between the normative thresholds relates to the outcome of interest could be missed.

The association of SGA, with motor, cognitive, and social developmental concerns at age 2 to 4 years, has been previously shown [10,11,35]. These studies categorised birthweight centile using traditional thresholds (<10th centile) or included a heterogeneous group of term and preterm babies and prematurity with its associated implications may have confounded the relationship. We have shown that lower birthweight centiles from 37 weeks of gestation, even within the accepted normal range, are associated with developmental concerns.

The association of LGA in term infants with neurodevelopmental concerns has been inconsistent [10,35–37]. Frank and colleagues [37], analysing 1,685 children, suggested that LGA, defined as birthweight >90th percentile, are not at increased risk for poor verbal ability or externalizing behaviour problems at 4 to 5 years. We found in a substantially larger cohort of children that birthweight ≥75th centile was not importantly associated with developmental concerns, before and after adjustment for confounders in both complete case and imputed datasets.

The analysis examining potential mediation including mode of delivery, use of analgesia and anaesthesia in labour, 5-minute Apgar score, and NNU admission, showed a marginal attenuation in relative risks. However, the associations of low birthweight centiles with developmental concerns remained significant, suggesting that the relationship was not driven by peripartum or immediate postpartum events. However, we acknowledge that we did not have any information on childhood exposures, e.g., early growth trajectories, that may also have mediated the associations. There is evidence to suggest that most SGA babies have caught up growth by age 2 years with about 10 percent remaining small through adulthood [38–41]. The first 2 years of life is a period with significant brain plasticity [42] and may provide opportunity to improve neurodevelopment. Although an earlier trial involving term SGA failed to demonstrate that faster weight gain confers neurodevelopmental benefits [43], more recent evidence suggests there could be some benefits [38].

The strength of this study lies in its large sample size and contemporary cohort of births over a 12-year period. The inclusion of all eligible deliveries minimises the risk of selection bias and data quality control measures strengthen the veracity of our inferences. We acknowledge that we were unable to control for parental factors, in particular parental education, which could impact on childhood development, however, adjusting for smoking, deprivation index, and substance misuse may capture some of the confounding due to these traits. The main focus of our analyses was on singleton infants born from 37 weeks of gestation and we acknowledge that exclusion of those born preterm and from multiple pregnancies may introduce selection bias if any factors related to preterm or multiple-birth influence early childhood development [44]. The consistency of our findings in the whole birth population, including preterm babies, provides reassurance against substantial selection bias. We recognise that the ASQ as a screening tool will over-diagnose developmental concerns when compared to more formal, objective assessments such as the Bayley Scales of Infant Development [45]. We appreciate that the subjective nature of the assessments and the difficulties assessing young children, particularly for speech, may have contributed to the strength of the associations observed for each parameter, however, training of health visitors and use of standardised methodology for assessment reduce the subjectivity. We acknowledge that we cannot assume causality between birthweight centiles and early childhood developmental concerns because there may be residual confounding factors, such as those relating to childhood illness and upbringing, for which data were not available. Similarly, we may need to interpret the findings of the mediational model with caution, especially because of the categorical nature of the selected mediators; however, our analysis reasonably satisfied other underpinning assumptions, i.e., that there be no measurement error in the mediator and that the dependent variables do not cause the mediator [30]. We recognised high missing data, especially in the earlier years of the birth cohort (2003 to 2007), which a previous audit has suggested was due to failure of parents to engage with the Child Health Surveillance Programme and loss of data along the data return pathway [46]. The sensitivity analyses and similarity in findings from complete case and imputed data analyses give additional reassurance that we could expect consistency in the findings. We appreciate that we could not offer more information on other growth characteristics that could differentiate constitutionally small from growth-restricted offspring. We anticipate that the magnitude of the associations will be even larger for growth-restricted babies as the effect will not be diluted by the number of the constitutionally small babies. We have not estimated the disparity between estimated fetal weight (EFW) and birthweight; however, this disparity has been summarised to be about 5% [47] that render our findings relevant when counselling women with expected birthweight in the lower centiles.

Findings from our study reiterate the need for evaluation of the existing thresholds of birthweight centiles used clinically. Since birthweight is closely related to EFW, our study suggests that EFW <25th centile might be used as a marker to identify fetuses at risk of long-term adverse outcomes. Birthweight centile less than the 25th centile can be used by health care workers (such as paediatricians, health visitors, and child health nurses) as additional risk “flag” for early childhood developmental concerns and to highlight to parents children who may need added monitoring and support to achieve their full developmental potential. Though, we acknowledge that this would require appropriate intervention development and testing in randomised control trials to establish public health and economic effect.

Key areas for future research should be using objective assessment tools, such as the Bayley Scales, to examine the relationship between birthweight centiles and development among singleton infants born from 37 weeks of gestation and exploring the impact of birthweight centiles on longer-term outcomes, such as school performance. In addition, balanced against any potential problems that could associated with earlier delivery, future research should explore whether earlier delivery of babies from 37 weeks of gestation with mild to moderate SGA, above the traditional thresholds for smallness, would minimise the in-utero exposure to factors contributing to the suboptimal growth and subsequently reduce impact on child’s development.

Our study showed a progressive increase in the risk of child developmental concerns from birthweight <25th centile for babies born from 37 weeks of gestation. Our findings strengthen the suggestion for reconsideration of the traditional birthweight thresholds used clinically and suggest consideration of early childhood monitoring and support measures for infants born from 37 weeks of gestation with lower birthweight centiles to potentially reduce the risk of developmental concerns in childhood.

## Supporting information

S1 STROBE Checklist STROBE Statement Checklist. (DOCX)

S1 Table Plausible ranges and categorisation of variables. BMI, body mass index; CS, cesarean section; NNU, neonatal unit; SIMD, Scottish Index of Multiple Deprivation.
(DOCX)

S2 Table Population birthweight centiles and approximated actual birthweight of study infants (in grams). §—*n* = 686,284; €—*n* = 295,200; ¥—*n* = 727,002; ¶—*n* = 40,718.
(DOCX)

S3 Table Variable missingness. ¥—All infants born from 37 weeks of gestation (37+0 to 43^+6^), *n* = 686,284. €—whole birth population, including preterm (28^+0^ to 43^+6^), *n* = 727,002. Data presented as n (%). BMI, body mass index; NNU, neonatal unit; SIMD, Scottish Index of Multiple Deprivation.
(DOCX)

S4 Table Characteristics of infants born from 37 weeks of gestation with and without missing outcomes. n (%)–Percentage presented in columns. BMI, body mass index; NNU, neonatal unit; SIMD, Scottish Index of Multiple Deprivation.
(DOCX)

S5 Table Predicting missingness of outcome variables for whole birth population^€^. €—Inclusive of preterm births (gestational ages 28^+0^ to 43^+6^ weeks), *n* = 727,002. §–Analysis was adjusted for maternal age, BMI, parity, year of birth, gestational age at delivery, child’s sex, smoking, substance misuse in pregnancy, alcohol intake, socioeconomic status, ethnicity, diabetes, pre-eclampsia, maternal infection during pregnancy, history of stillbirth and spontaneous abortion, induction of labour, mode of delivery, use of analgesia/anaesthesia in labour, Apgar score at 5 minutes, and neonatal unit admission. ¥–Analysis was adjusted for birthweight, maternal age, parity, year of birth, child’s sex, smoking, substance misuse in pregnancy, alcohol intake, socioeconomic status, ethnicity, diabetes, pre-eclampsia, maternal infection during pregnancy, history of stillbirth and spontaneous abortion, induction of labour, mode of delivery, use of analgesia/anaesthesia in labour, Apgar score at 5 minutes, and neonatal unit admission. ₣–Analysis was adjusted for birthweight, maternal age, BMI, parity, gestational age at delivery, child’s sex, smoking, substance misuse in pregnancy, alcohol intake, socioeconomic status, ethnicity, diabetes, pre-eclampsia, maternal infection during pregnancy, history of stillbirth and spontaneous abortion, induction of labour, mode of delivery, use of analgesia/anaesthesia in labour, Apgar score at 5 minutes, and neonatal unit admission.
(DOCX)

S6 Table The proportion of children by birthweight centiles with developmental concern in each domain (gestational age 37^+0^ to 43^+6^). §: *n* = 295,200. ¥: *n* = 118,325. Adjusted for maternal age, BMI, parity, year of birth, gestational age at delivery, child’s sex, smoking, illicit drug use in pregnancy, alcohol intake, socioeconomic status, ethnicity, diabetes, pre-eclampsia, maternal infection during pregnancy, history of stillbirth and spontaneous abortion, and induction of labour.
(DOCX)

S7 Table Adjusted RRs of developmental concerns of imputed data (all infants born from 37 weeks of gestation). –*n* = 686,284. Analysis was adjusted for maternal age, BMI, parity, year of birth, gestational age at delivery, child’s sex, smoking, substance misuse in pregnancy, alcohol intake, socioeconomic status, ethnicity, diabetes, pre-eclampsia, maternal infection during pregnancy, history of stillbirth and spontaneous abortion, and induction of labour.
(DOCX)

S8 Table Adjusted RR of developmental concerns after adjusting for potential mediators and child’s age at developmental assessment (for gestational age 37^+0^ to 43^+6^). ¥—*n* = 113,794, analysis was adjusted for confounders (maternal age, BMI, parity, year of birth, gestational age at delivery, child’s sex, smoking, substance misuse in pregnancy, alcohol intake, socioeconomic status, ethnicity, diabetes, pre-eclampsia, maternal infection during pregnancy, history of stillbirth and spontaneous abortion, and induction of labour) and potential mediators (mode of delivery, use of analgesia/anaesthesia in labour, Apgar score at 5 minutes, and special baby care unit admission). ₣—*n* = 113,794, analysis was adjusted for confounders (maternal age, BMI, parity, year of birth, gestational age at delivery, child’s sex, smoking, substance misuse in pregnancy, alcohol intake, socioeconomic status, ethnicity, diabetes, pre-eclampsia, maternal infection during pregnancy, history of stillbirth and spontaneous abortion, and induction of labour), potential mediators (mode of delivery, use of analgesia/anaesthesia in labour, Apgar score at 5 minutes, and special baby care unit admission), and child’s age at developmental assessment.
(DOCX)

S9 Table RRs of developmental concerns of SGA (<2,500 g) and LGA (>4,000 g) referent to birthweight between 2,500 g and 4,000 g (for gestational age 37^+0^ to 43^+6^). §–Unadjusted, CCA, *n* = 295,200. ¥—*n* = 118,325. Analysis was adjusted for **confounders** (maternal age, BMI, parity, year of birth, gestational age at delivery, child’s sex, smoking, substance misuse in pregnancy, alcohol intake, socioeconomic status, ethnicity, diabetes, pre-eclampsia, maternal infection during pregnancy, history of stillbirth and spontaneous abortion, and induction of labour). ¶—*n* = 113,794. Analysis adjusted for **confounders** (maternal age, BMI, parity, year of birth, gestational age at delivery, child’s sex, smoking, substance misuse in pregnancy, alcohol intake, socioeconomic status, ethnicity, diabetes, pre-eclampsia, maternal infection during pregnancy, history of stillbirth and spontaneous abortion, and induction of labour) and **potential mediators** (mode of delivery, use of analgesia/anaesthesia in labour, Apgar score at 5 minutes, and special baby care unit admission).
(DOCX)

S10 Table RRs of developmental concerns using more granular absolute birthweight categories (gestational age 37^+0^ to 43^+6^). §–Unadjusted, CCA, *n* = 295,200. ¥—*n* = 118,325. Analysis was adjusted for **confounders** (maternal age, BMI, parity, year of birth, gestational age at delivery, child’s sex, smoking, substance misuse in pregnancy, alcohol intake, socioeconomic status, ethnicity, diabetes, pre-eclampsia, maternal infection during pregnancy, history of stillbirth and spontaneous abortion, and induction of labour). ¶—*n* = 113,794. Analysis adjusted for **confounders** (maternal age, BMI, parity, year of birth, gestational age at delivery, child’s sex, smoking, substance misuse in pregnancy, alcohol intake, socioeconomic status, ethnicity, diabetes, pre-eclampsia, maternal infection during pregnancy, history of stillbirth and spontaneous abortion, and induction of labour) and **potential mediators** (mode of delivery, use of analgesia/anaesthesia in labour, Apgar score at 5 minutes, and special baby care unit admission).
(DOCX)

S11 Table The proportion of children by birthweight centiles with developmental concern in each domain (gestational age 37^+0^ to 41^+6^). §: *n* = 287,414. ¥: *n* = 115,314. Adjusted for maternal age, BMI, parity, year of birth, gestational age at delivery, child’s sex, smoking, illicit drug use in pregnancy, alcohol intake, socioeconomic status, ethnicity, diabetes, pre-eclampsia, maternal infection during pregnancy, history of stillbirth and spontaneous abortion, and induction of labour.
(DOCX)

S12 Table RRs of developmental concerns for gestational age 37^+0^ to 41^+6^. §–Unadjusted, CCA, *n* = 287,414. ¥—*n* = 115,314. Analysis was adjusted for **confounders** (maternal age, BMI, parity, year of birth, gestational age at delivery, child’s sex, smoking, substance misuse in pregnancy, alcohol intake, socioeconomic status, ethnicity, diabetes, pre-eclampsia, maternal infection during pregnancy, history of stillbirth and spontaneous abortion, and induction of labour). ¶—*n* = 110,877. Analysis adjusted for **confounders** (maternal age, BMI, parity, year of birth, gestational age at delivery, child’s sex, smoking, substance misuse in pregnancy, alcohol intake, socioeconomic status, ethnicity, diabetes, pre-eclampsia, maternal infection during pregnancy, history of stillbirth and spontaneous abortion, and induction of labour) and **potential mediators** (mode of delivery, use of analgesia/anaesthesia in labour, Apgar score at 5 minutes, and special baby care unit admission).
(DOCX)

S13 Table RRs of developmental concerns for whole birth population (infants born from 28^+0^ to 43^+6^). §–Unadjusted, CCA, *n* = 309,193. ¥—Adjusted CCA, *n* = 122,263. Analysis was adjusted for maternal age, BMI, parity, year of birth, gestational age at delivery, child’s sex, smoking, substance misuse in pregnancy, alcohol intake, socioeconomic status, ethnicity, diabetes, pre-eclampsia, maternal infection during pregnancy, history of stillbirth and spontaneous abortion, and induction of labour. ¶–Adjusted analysis, imputed data, *n* = 727,002. Analysis was adjusted for same covariates as in CCA.
(DOCX)

S14 Table Check for variance in outcome domains within 25th and 74th birthweight centiles (for gestational age 37^+0^ to 43^+6^). §–Unadjusted, *n* = 147,789. ¥—Adjusted, *n* = 59,977. Analysis was adjusted for maternal age, BMI, parity, year of birth, gestational age at delivery, child’s sex, smoking, substance misuse in pregnancy, alcohol intake, socioeconomic status, ethnicity, diabetes, pre-eclampsia, maternal infection during pregnancy, history of stillbirth and spontaneous abortion, and induction of labour.
(DOCX)

S15 Table PAFs of infants born from 37 weeks gestational age babies within the whole birth population (28^+0^ to 43^+6^ weeks). ¥—Adjustment for maternal age, BMI, parity, year of birth, gestational age at delivery, child’s sex, smoking, substance misuse in pregnancy, alcohol intake, socioeconomic status [deprivation index], ethnicity, diabetes, pre-eclampsia, maternal infection during pregnancy, history of stillbirth and spontaneous abortion, and induction of labour, referent to birthweight between 25th and 74th centile. Result presented as percent (95% CI).
(DOCX)

## Abbreviations

BMI: body mass index
CHI: Community Health Index
EFW: estimated fetal weight
LBW: low birthweight
LGA: large for gestational age
MICE: multiple imputation by chained equation
NNU: neonatal unit
PAF: population attributable fraction
RR: relative risk
SGA: small for gestational age
SIMD: Scottish Index of Multiple Deprivation
STI: sexually transmitted infection

## References

[1] Gill SV, May-Benson TA, Teasdale A, Munsell EG. Birth and developmental correlates of birth weight in a sample of children with potential sensory processing disorder. BMC Pediatr. 2013;13(1):29. Epub 2013/02/28. doi: 10.1186/1471-2431-13-29 . 23442948 PMC3598529

[2] Kirk CM, Uwamungu JC, Wilson K, Hedt-Gauthier BL, Tapela N, Niyigena P, et al. Health, nutrition, and development of children born preterm and low birth weight in rural Rwanda: a cross-sectional study. BMC Pediatr. 2017;17(1):191. Epub 2017/11/17. doi: 10.1186/s12887-017-0946-1 . 29141590 PMC5688768

[3] Ross G, Demaria R, Yap V. The Relationship Between Motor Delays and Language Development in Very Low Birthweight Premature Children at 18 Months Corrected Age. J Speech Lang Hear Res. 2018;61(1):114–9. Epub 2017/12/20. doi: 10.1044/2017_JSLHR-L-17-0056 . 29255850

[4] Ribeiro CD, Pachelli MR, Amaral NC, Lamônica DA. Development skills of children born premature with low and very low birth weight. Codas. 2017;29(1):e20160058. Epub 2017/02/02. doi: 10.1590/2317-1782/20162016058 . 28146204

[5] Zerbeto AB, Cortelo FM, Filho ÉBC. Association between gestational age and birth weight on the language development of Brazilian children: a systematic review. J Pediatr (Rio J). 2015;91(4):326–32. Epub 2015/04/29. doi: 10.1016/j.jped.2014.11.003 . 25913048

[6] Thompson JR, Carter RL, Edwards AR, Roth J, Ariet M, Ross NL, et al. A population-based study of the effects of birth weight on early developmental delay or disability in children. Am J Perinatol. 2003;20(6):321–32. Epub 2003/10/07. doi: 10.1055/s-2003-42773 . 14528402

[7] Madigan S, Wade M, Plamondon A, Browne D, Jenkins JM. Birth Weight Variability and Language Development: Risk, Resilience, and Responsive Parenting. J Pediatr Psychol. 2015;40(9):869–77. Epub 2015/06/27. doi: 10.1093/jpepsy/jsv056 . 26112158

[8] Boulet SL, Schieve LA, Boyle CA. Birth weight and health and developmental outcomes in US children, 1997–2005. Matern Child Health J. 2011;15(7):836–44. Epub 2009/11/11. doi: 10.1007/s10995-009-0538-2 . 19902344

[9] Koyanagi A, Zhang J, Dagvadorj A, Hirayama F, Shibuya K, Souza JP, et al. Macrosomia in 23 developing countries: an analysis of a multicountry, facility-based, cross-sectional survey. Lancet. 2013;381(9865):476–483. doi: 10.1016/S0140-6736(12)61605-5 23290494

[10] Moore GS, Kneitel AW, Walker CK, Gilbert WM, Xing G. Autism risk in small-and large-for-gestational-age infants. Am J Obstet Gynecol. 2012;206(4):314.e1–.e9. doi: 10.1016/j.ajog.2012.01.044 22464070 PMC9884028

[11] Takeuchi A, Yorifuji T, Takahashi K, Nakamura M, Kageyama M, Kubo T, et al. Neurodevelopment in full-term small for gestational age infants: A nationwide Japanese population-based study. Brain Dev. 2016;38(6):529–537. doi: 10.1016/j.braindev.2015.12.013 26791811

[12] Iliodromiti S, Mackay DF, Smith GC, Pell JP, Sattar N, Lawlor DA, et al. Customised and noncustomised birth weight centiles and prediction of stillbirth and infant mortality and morbidity: a cohort study of 979,912 term singleton pregnancies in Scotland. PLoS Med. 2017;14(1):e1002228. doi: 10.1371/journal.pmed.1002228 28141865 PMC5283655

[13] Mackay D, Smith G, Dobbie R, Cooper SA, Pell J. Obstetric factors and different causes of special educational need: retrospective cohort study of 407 503 schoolchildren. BJOG. 2013;120(3):297–308. doi: 10.1111/1471-0528.12071 23189965

[14] Ages and Stages Questionnaire. ASQ-3 2021 [cited 2021 Nov 22]. https://agesandstages.com/.

[15] Scottish Government. The Scottish Child Health Programme: Guidance on the 27–30 month child health review. 2012 [cited 2021 Jul 7]. https://www.gov.scot/publications/scottish-child-health-programme-guidance-27-30-month-child-health-review/.

[16] Scottish Government. Child Health Surveillance Programme Pre-school Clinical Guidelines. 2018 [cited 2021]. https://www.isdscotland.org/Health-Topics/Child-Health/Child-Health-Programme/_docs/CHSP-PS-Clinical-Guidelines-2018-06-26-FINAL.pdf.

[17] Scottish Government. Universal Health Visiting Pathway in Scotland: pre-birth to pre-school. 2015 [cited 2021 Jul 7]. https://www.gov.scot/publications/universal-health-visiting-pathway-scotland-pre-birth-pre-school/.

[18] Age and Stages Questionnaires. Training Module: Interpreting Results and Next Steps. 2018 [cited 2021 Nov 21]. https://agesandstages.com/wp-content/uploads/2018/02/interpretingresults_slidepresentation_020818.pdf.

[19] Kearns RJ, Shaw M, Gromski PS, Iliodromiti S, Pell JP, Lawlor DA, et al. Neonatal and early childhood outcomes following maternal anesthesia for cesarean section: a population-based cohort study. Reg Anesth Pain Med. 2021. Epub 2021/04/10. doi: 10.1136/rapm-2020-102441 . 33832987

[20] Information Services Division. Child Health 27–30 Month Review Statistics, Scotland 2016/17. Technical report. Edinburgh, Scotland: NHS; 2018.

[21] MacKay DF, Smith GC, Dobbie R, Pell JP. Gestational age at delivery and special educational need: retrospective cohort study of 407,503 schoolchildren. PLoS Med. 2010;7(6):e1000289. doi: 10.1371/journal.pmed.1000289 20543995 PMC2882432

[22] Scottish Government. Scottish Index of Multiple Deprivation 2020. [cited 2020 July 13]. https://www.gov.scot/collections/scottish-index-of-multiple-deprivation-2020/.

[23] Scotland’s Census. Scotland’s census ethnic group classification 2011. [cited 2020 June 9]. https://www.scotlandscensus.gov.uk/census-results/at-a-glance/ethnicity/.

[24] Draper ES, Gallimore ID, Smith LK, Fenton AC, Kurinczuk JJ, Smith PW, et al. MBRRACE-UK Perinatal Mortality Surveillance Report, UK Perinatal Deaths for Births from January to December 2019. Leicester: The Infant Mortality and Morbidity Studies, Department of Health Sciences, University of Leicester, 2021.

[25] Royston P, Sauerbrei W. Multivariable modeling with cubic regression splines: a principled approach. Stata J. 2007;7(1):45–70.

[26] Greenland S, Rothman K, Lash T. Measures of effect and measures of association. In: Rothman K, Greenland S, Lash T, editors. Modern Epidemiology. Lippincott Williams & Wilkins; 2008. p. 51–70.

[27] Mansournia MA, Altman DG. Population attributable fraction. BMJ. 2018;360:k757. doi: 10.1136/bmj.k757 29472187

[28] Chojnacki MR, Holscher HD, Balbinot AR, Raine LB, Biggan JR, Walk AM, et al. Relations between mode of birth delivery and timing of developmental milestones and adiposity in preadolescence: A retrospective study. Early Hum Dev. 2019;129:52–9. Epub 2019/01/15. doi: 10.1016/j.earlhumdev.2018.12.021 . 30641478 PMC6382526

[29] Subedi D, DeBoer MD, Scharf RJ. Developmental trajectories in children with prolonged NICU stays. Arch Dis Child. 2017;102(1):29–34. doi: 10.1136/archdischild-2016-310777 27637907

[30] Baron RM, Kenny DA. The moderator–mediator variable distinction in social psychological research: Conceptual, strategic, and statistical considerations. J Pers Soc Psychol. 1986;51(6):1173. doi: 10.1037//0022-3514.51.6.1173 3806354

[31] Royston P, White IR. Multiple imputation by chained equations (MICE): implementation in Stata. J Stat Softw. 2011;45(4):1–20.

[32] Raghunathan TE, Solenberger PW, Van Hoewyk J. IVEware: Imputation and Variance Estimation Software. Ann Arbor, MI: Survey Research Center, Institute for Social Research, University of Michigan; 2002.

[33] Hughes RA, Heron J, Sterne JAC, Tilling K. Accounting for missing data in statistical analyses: multiple imputation is not always the answer. Int J Epidemiol. 2019;48(4):1294–304. Epub 2019/03/18. doi: 10.1093/ije/dyz032 . 30879056 PMC6693809

[34] Chen R, Tedroff K, Villamor E, Lu D, Cnattingius S. Risk of intellectual disability in children born appropriate-for-gestational-age at term or post-term: impact of birth weight for gestational age and gestational age. Eur J Epidemiol. 2020;35(3):273–282. doi: 10.1007/s10654-019-00590-7 31788734 PMC7154017

[35] Tamai K, Yorifuji T, Takeuchi A, Fukushima Y, Nakamura M, Matsumoto N, et al. Associations of Birth Weight for Gestational Age with Child Health and Neurodevelopment among Term Infants: A Nationwide Japanese Population-Based Study. J Pediatr. 2020;226:135–41.e4. doi: 10.1016/j.jpeds.2020.06.075 32640270

[36] Lei X, Zhao D, Huang L, Luo Z, Zhang J, Yu X, et al. Childhood health outcomes in term, large-for-gestational-age babies with different postnatal growth patterns. Am J Epidemiol. 2018;187(3):507–514. doi: 10.1093/aje/kwx271 28992219

[37] Frank CE, Speechley KN, Macnab JJ, Campbell MK. Infants born large for gestational age and developmental attainment in early childhood. Int J Pediatr. 2018;2018:9181497. doi: 10.1155/2018/9181497 29535788 PMC5817806

[38] Taine M, Forhan A, Morgan AS, Bernard JY, Peyre H, Dufourg MN, et al. Early postnatal growth and subsequent neurodevelopment in children delivered at term: The ELFE cohort study. Paediatr Perinat Epidemiol. 2021;35(6):748–757. doi: 10.1111/ppe.12798 34255382

[39] Ong KK. Catch-up growth in small for gestational age babies: good or bad? Curr Opin Endocrinol Diabetes Obes. 2007;14(1):30–34. doi: 10.1097/MED.0b013e328013da6c 17940416

[40] Cho WK, Suh B-K. Catch-up growth and catch-up fat in children born small for gestational age. Korean J Pediatr. 2016;59(1):1–7. Epub 2016/01/22. doi: 10.3345/kjp.2016.59.1.1 . 26893597 PMC4753194

[41] Argente J, Mehls O, Barrios V. Growth and body composition in very young SGA children. Pediatr Nephrol. 2010;25(4):679–685. doi: 10.1007/s00467-009-1432-2 20108001

[42] Gluckman P, Hanson M. Developmental plasticity and human disease: research directions. J Intern Med. 2007;261(5):461–471. doi: 10.1111/j.1365-2796.2007.01802.x 17444885

[43] Morley R, Fewtrell MS, Abbott R A, Stephenson T, MacFadyen U, Lucas A. Neurodevelopment in children born small for gestational age: a randomized trial of nutrient-enriched versus standard formula and comparison with a reference breastfed group. Pediatrics. 2004;113(3):515–521. doi: 10.1542/peds.113.3.515 14993543

[44] Pearce N, Lawlor DA. Causal inference—so much more than statistics. Int J Epidemiol. 2016;45(6):1895–1903. doi: 10.1093/ije/dyw328 28204514 PMC5841844

[45] Bayley N. Bayley scales of infant and toddler development–Third Edition: Technical manual. San Antonio, TX: The Psychological Corporation; 2006.

[46] Wood R, Stirling A. Audit of children with no CHSP-PS record of a health visitor first visit and/or 6–8 week review. Scotland: Information Services Division (ISD); 2012.

[47] Milner J, Arezina J. The accuracy of ultrasound estimation of fetal weight in comparison to birth weight: A systematic review. Ultrasound. 2018;26(1):32–41. doi: 10.1177/1742271X17732807 29456580 PMC5810856

