# Supplementary material for: Association of birthweight centiles and early childhood development of singleton infants born from 37 weeks of gestation in Scotland: A population-based cohort study
Source: PLoS Med. 2022 Oct 11;19(10):e1004108. doi: 10.1371/journal.pmed.1004108 (PMC9553050; doi:10.1371/journal.pmed.1004108)
Supplement: S1 STROBE Checklist — (DOCX) [file pmed.1004108.s001.docx]

S1 STROBE Checklist. STROBE Statement Checklist

|  | **Item No** | **Recommendation** |
| --- | --- | --- |
| **Title and abstract** | 1 √  √ | (*a*) Indicate the study’s design with a commonly used term in the title or the abstract (Title page) |
|  |  | (*b*) Provide in the abstract an informative and balanced summary of what was done and what was found (Abstract - Method and Findings) |
| **Introduction** | | |
| Background/rationale | 2 √ | Explain the scientific background and rationale for the investigation being reported (Introduction, paragraphs 1 and 2) |
| Objectives | 3 √ | State specific objectives, including any prespecified hypotheses (Introduction, paragraph 2) |
| **Methods** | | |
| Study design | 4 √ | Present key elements of study design early in the paper (Method, study designs and population subsection - paragraph 1) |
| Setting | 5 √ | Describe the setting, locations, and relevant dates, including periods of recruitment, exposure, follow-up, and data collection (Method, study designs and population subsection - paragraph 2 and 3) |
| Participants | 6 √ | (*a*) Give the eligibility criteria, and the sources and methods of selection of participants. Describe methods of follow-up (Method, study designs and population subsection - paragraph 4) |
|  |  | (*b*) For matched studies, give matching criteria and number of exposed and unexposed (Not relevant to study design as not matched study) |
| Variables | 7 √ | Clearly define all outcomes, exposures, predictors, potential confounders, and effect modifiers. Give diagnostic criteria, if applicable (Methods - outcome, exposure, and covariates subsections) |
| Data sources/ measurement | 8* √ | For each variable of interest, give sources of data and details of methods of assessment (measurement). Describe comparability of assessment methods if there is more than one group (Method, study designs and population subsection - paragraph 1, 2 and 3; Table S1) |
| Bias | 9 √ | Describe any efforts to address potential sources of bias (Method, missing data subsection) |
| Study size | 10 √ | Explain how the study size was arrived at (Method, study designs and population subsection - paragraph 1; Results, paragraph 1, Figure 1) |
| Quantitative variables | 11 √ | Explain how quantitative variables were handled in the analyses. If applicable, describe which groupings were chosen and why (Methods - outcome, exposure, and covariates subsections, Table S1) |
| Statistical methods | 12 √ | (*a*) Describe all statistical methods, including those used to control for confounding (Method, statistical analyses subsection, supplementary analyses subsection |
|  |  | (*b*) Describe any methods used to examine subgroups and interactions (Method, supplementary analyses subsection) |
|  |  | (*c*) Explain how missing data were addressed (Method, missing data subsection) |
|  |  | (*d*) If applicable, explain how loss to follow-up was addressed (NA) |
|  |  | (*e*) Describe any sensitivity analyses (Method, supplementary analyses subsection) |
| **Results** | | |
| Participants | 13 √ | (a) Report numbers of individuals at each stage of study—eg numbers potentially eligible, examined for eligibility, confirmed eligible, included in the study, completing follow-up, and analysed (Results, paragraph 1) |
|  |  | (b) Give reasons for non-participation at each stage (Figure 1) |
|  |  | (c) Consider use of a flow diagram (Figure 1) |
| Descriptive data | 14* √ | (a) Give characteristics of study participants (eg demographic, clinical, social) and information on exposures and potential confounders (Results, paragraph 2; Tables 1) |
|  |  | (b) Indicate number of participants with missing data for each variable of interest (Table S3) |
|  |  | (c) Summarise follow-up time (eg, average and total amount) (Results, paragraph 1) |
| Outcome data | 15* √ | Report numbers of outcome events or summary measures over time (Result, paragraph 1; Table 1) |
| Main results | 16 √ | (*a*) Give unadjusted estimates and, if applicable, confounder-adjusted estimates and their precision (eg, 95% confidence interval). Make clear which confounders were adjusted for and why they were included (Results, paragraphs 4 to 7; Table 2 and Tables S6 to S11) |
|  |  | (*b*) Report category boundaries when continuous variables were categorized (Table S1) |
|  |  | (*c*) If relevant, consider translating estimates of relative risk into absolute risk for a meaningful time period |
| Other analyses | 17 √ | Report other analyses done—eg analyses of subgroups and interactions, and sensitivity analyses (Results, paragraphs 5 to 7, Tables S6 to S11) |
| **Discussion** | | |
| Key results | 18 √ | Summarise key results with reference to study objectives (Discussion, main findings subsection) |
| Limitations | 19 √ | Discuss limitations of the study, taking into account sources of potential bias or imprecision. Discuss both direction and magnitude of any potential bias (Discussion, strengths and limitations subsection) |
| Interpretation | 20 √ | Give a cautious overall interpretation of results considering objectives, limitations, multiplicity of analyses, results from similar studies, and other relevant evidence Discussion, comparison with existing studies subsection) |
| Generalisability | 21 √ | Discuss the generalisability (external validity) of the study results (Discussion, clinical implication subsection) |
| **Other information** | | |
| Funding | 22 | Give the source of funding and the role of the funders for the present study and, if applicable, for the original study on which the present article is based (under Funding) |

*Give information separately for non-imputed and imputed data.
