# Supplementary material for: Association of birthweight centiles and early childhood development of singleton infants born from 37 weeks of gestation in Scotland: A population-based cohort study
Source: PLoS Med. 2022 Oct 11;19(10):e1004108. doi: 10.1371/journal.pmed.1004108 (PMC9553050; doi:10.1371/journal.pmed.1004108)
Supplement: S1 Table — BMI, body mass index; CS, cesarean section; NNU, neonatal unit; SIMD, Scottish Index of Multiple Deprivation. (DOCX) [file pmed.1004108.s002.docx]

S1 Table. Plausible ranges and categorization of variables

| **Variables** | **Type** | **Plausible ranges/categorization** |
| --- | --- | --- |
| Fine motor | Categorical | Concern \| No concern |
| Gross motor | Categorical | Concern \| No concern |
| Communication | Categorical | Concern \| No concern |
| Social skills | Categorical | Concern \| No concern |
| Birthweight | Continuous | Truncated at < 500g and > 7000g |
| Maternal age | Discrete | Truncated at age < 10 years |
| Parity | Discrete | Nulliparity \| Multiparity |
| Maternal weight | Continuous | Truncated at < 30kg and > 300kg |
| Maternal height | Continuous | Truncated at <100cm and >200cm |
| Maternal BMI (kg/m^2^) | Ordinal | <18.5 \| 18.5-24.9 \|25.0-29.9\|30.0-34.9 \|≥35.0 |
| Year of birth | Ordinal | 2003 to 2015 (grouped in 4-year category) |
| Gestational age (term) | Continuous | ≥37^+0^ to 43^+6^ |
| Smoking history | Categorical | Never \| Former \| Current smoker |
| Substance misuse in pregnancy | Categorical | No \| Yes |
| Weekly alcohol intake | Discrete | None \| 1-2 units \| ≥3 units |
| SIMD Decile | Ordinal | 1 to 10 |
| Ethnicity | Categorical | White \| Asian \| Black \| Mixed & other |
| Diabetes | Categorical | No \| Yes |
| Pre-eclampsia | Categorical | No \| Yes |
| Maternal infection during pregnancy | Categorical | No \| Yes |
| Previous history of stillbirth | Categorical | No \| Yes |
| Previous spontaneous abortion | Discrete | Count |
| Induction of labour | Categorical | No \| Yes |
| Mode of delivery | Categorical | Spontaneous cephalic \| Instrumental cephalic \| Assisted Vaginal breech \| Elective CS \| Emergency CS |
| Analgesia in labour | Categorical | Opiates \| Gas and air only \| Spinal (incl. combined) \| General anaesthesia |
| Apgar score at 5 minutes | Discrete | < 7 \| ≥7 |
| NNU admission | Categorical | Not admitted \| Admitted (up to 48 hours) \| Admitted (beyond 48 hours) |

BMI – Body Mass Index; SIMD – Scottish Index of Multiple Deprivation; CS – Caesarean Section; NNU – Neonatal Unit
