# Supplementary material for: Association of birthweight centiles and early childhood development of singleton infants born from 37 weeks of gestation in Scotland: A population-based cohort study
Source: PLoS Med. 2022 Oct 11;19(10):e1004108. doi: 10.1371/journal.pmed.1004108 (PMC9553050; doi:10.1371/journal.pmed.1004108)
Supplement: S2 Table — §—n = 686,284; €—n = 295,200; ¥—n = 727,002; ¶—n = 40,718. (DOCX) [file pmed.1004108.s003.docx]

S2 Table. Population birthweight centiles and approximated actual birthweight of study infants (in grams)

| **Population**  **Birthweight centiles** | **Birthweights (in grams)** | | | |
| --- | --- | --- | --- | --- |
|  | **All infants born from 37weeks ^§^** | **Infants born from 37 weeks with follow-up data ^€^** | **Whole birth population (including preterm) ^¥^** | **Preterm only** **^¶^** |
| 1% | 2330 | 2310 | 1805 | 980 |
| 5% | 2690 | 2670 | 2495 | 1320 |
| 10% | 2870 | 2850 | 2750 | 1560 |
| 25% | 3160 | 3142 | 3100 | 2010 |
| 50% | 3480 | 3460 | 3440 | 2440 |
| 75% | 3800 | 3800 | 3780 | 2800 |
| 90% | 4110 | 4100 | 4100 | 3140 |
| 95% | 4300 | 4300 | 4290 | 3375 |
| 99% | 4680 | 4680 | 4678 | 3920 |

**^§^ -** n=686,284; **^€^** - n=295,200; **^¥^ -** n= 727,002; ^¶^ - n=40,718
