# Supplementary material for: Association of birthweight centiles and early childhood development of singleton infants born from 37 weeks of gestation in Scotland: A population-based cohort study
Source: PLoS Med. 2022 Oct 11;19(10):e1004108. doi: 10.1371/journal.pmed.1004108 (PMC9553050; doi:10.1371/journal.pmed.1004108)
Supplement: S3 Table — ¥—All infants born from 37 weeks of gestation (37+0 to 43+6), n = 686,284. €—whole birth population, including preterm (28+0 to 43+6), n = 727,002. Data presented as n (%). BMI, body mass index; NNU, neonatal unit; SIMD, Scottish Index of Multiple Deprivation. (DOCX) [file pmed.1004108.s004.docx]

S3 Table. Variable missingness

| Variable | Main study population only ^¥^ | Main study population + preterm births ^€^ |
| --- | --- | --- |
| Fine motor | 385,094 (56.11) | 406,371 (55.90) |
| Gross motor | 382,883 (55.79) | 403,961 (55.57) |
| Communication | 381,926 (55.65) | 403,080 (55.44) |
| Social skills | 381,272 (55.56) | 402,334 (55.34) |
| Maternal BMI | 193,144 (28.14) | 233,862 (32.17) |
| Parity | 4,837 (0.70) | 5,216 (0.72) |
| Year of delivery | 45,827 (6.68) | 48,771 (6.71) |
| Smoking history | 35,400 (5.16) | 38,253 (5.26) |
| Substance misuse in pregnancy | 328,805 (47.91) | 348,463 (47.93) |
| Weekly alcohol intake | 321,092 (46.79) | 340,386 (46.82) |
| SIMD Decile | 1,176 (0.17) | 1,244 (0.17) |
| Ethnicity | 400,647 (58.38) | 423,910 (58.31) |
| Previous history of stillbirth | 1,862 (0.27) | 1,996 (0.27) |
| Previous spontaneous abortion | 1,378 (0.20) | 1,486 (0.20) |
| Induction of labour | 3,844 (0.56) | 4,116 (0.57) |
| Mode of delivery | 98,025 (14.28) | 111,710 (15.37) |
| Use of analgesia/anaesthesia in labour | 110,085 (16.04) | 116,837 (16.07) |
| Apgar score at 5 minutes | 12,391 (1.81) | 13,981 (1.92) |
| NNU admission | 11,871 (1.73) | 12,635 (1.74) |
| Child’s sex | 0 (0) | 0 (0) |
| Estimated gestational age | 0 (0) | 0 (0) |
| Birthweight | 0 (0) | 0 (0) |
| Maternal age | 0 (0) | 0 (0) |
| Maternal medical conditions (e.g., diabetes, pre-eclampsia) | 0 (0) | 0 (0) |

BMI – Body Mass Index, SIMD – Scottish Index of Multiple Deprivation; NNU – Neonatal Unit

^¥^ - all infants born from 37 weeks of gestation (37^+0^ to 43^+6^), n=686,284

^€^ - whole birth population, including preterm (28^+0^ to 43^+6^), n= 727,002

Data presented as n (%).
