# Supplementary material for: Association of birthweight centiles and early childhood development of singleton infants born from 37 weeks of gestation in Scotland: A population-based cohort study
Source: PLoS Med. 2022 Oct 11;19(10):e1004108. doi: 10.1371/journal.pmed.1004108 (PMC9553050; doi:10.1371/journal.pmed.1004108)
Supplement: S4 Table — n (%)–Percentage presented in columns. BMI, body mass index; NNU, neonatal unit; SIMD, Scottish Index of Multiple Deprivation. (DOCX) [file pmed.1004108.s005.docx]

S4 Table. Characteristics of infants born from 37 weeks of gestation with and without missing outcomes.

| **Variable** | **Frequency, n** | **Missing outcome?** | |
| --- | --- | --- | --- |
|  |  | *No* | *Yes* |
| **Maternal age** (years) | 686,284 | 28.79 ± 6.05 | 29.30 ± 5.92 |
| **Maternal BMI** (kg/m^2^) | 493,140 | 26.15 ± 5.68 | 26.11 ± 5.57 |
| **Parity** | 681,447 |  |  |
| Nulliparous |  | 130,371 (44.50) | 174,029 (44.80) |
| Multiparous |  | 162,597 (55.50) | 214,450 (55.20) |
| **Year of birth** | 640,457 |  |  |
| 2003-2007 |  | 74,667 (25.29) | 160,195 (46.40) |
| 2008-2011 |  | 79,423 (26.90) | 127,067 (36.80) |
| 2012-2015 |  | 141,110 (47.80) | 57,995 (16.80) |
| **Gestational age** (weeks) | 686,284 |  |  |
| 37^+0^ to 38^+6^ |  | 54,549 (18.48) | 71,382 (18.25) |
| 39^+0^ to 40^+6^ |  | 164,956 (55.88) | 218,374 (55.84) |
| 41^+0^ to 41^+6^ |  | 67,909 (23.00) | 90,277 (23.08) |
| 42^+0^ to 43^+6^ |  | 7,786 (2.64) | 11,051 (2.83) |
| **Smoking history** | 650,884 |  |  |
| Never smoked |  | 182,677 (64.83) | 256,549 (69.50) |
| Former smoker |  | 32,563 (11.56) | 38,964 (10.56) |
| Current smoker |  | 66,521 (23.61) | 73,610 (19.94) |
| **Substance misuse in pregnancy** | 357,479 |  |  |
| No |  | 187,069 (97.62) | 162,775 (98.14) |
| Yes |  | 4,553 (2.38) | 3,082 (1.86) |
| **Weekly alcohol intake** | 365,192 |  |  |
| None |  | 178,719 (93.97) | 160,517 (91.72) |
| 1-2 units |  | 3,655 (1.92) | 5,105 (2.92) |
| ≥3 units |  | 7,811 (4.11) | 9,385 (5.36) |
| **SIMD Decile** | 685,108 |  |  |
| 1 |  | 45,083 (15.29) | 47,259 (12.11) |
| 2 |  | 38,703 (13.13) | 44,342 (11.36) |
| 3 |  | 33,083 (11.22) | 41,090 (10.53) |
| 4 |  | 30,149 (10.23) | 38,602 (9.89) |
| 5 |  | 28,288 (9.59) | 37,011 (9.48) |
| 6 |  | 25,144 (8.53) | 34,948 (8.95) |
| 7 |  | 25,101 (8.51) | 35,615 (9.13) |
| 8 |  | 24,402 (8.28) | 37,688 (9.66) |
| 9 |  | 23,747 (8.05) | 36,907 (9.46) |
| 10 |  | 21,139 (7.17) | 36,807 (9.43) |
| **Induction of labour** | 682,440 |  |  |
| No |  | 214,856 (73.24) | 289,262 (74.35) |
| Yes |  | 78,514 (26.76) | 99,808 (25.65) |
| **Child’s sex** | 686,284 |  |  |
| Male |  | 150,844 (51.10) | 199,626 (51.04) |
| Female |  | 144,356 (48.90) | 191,458 (48.96) |
| **Apgar score (5 mins)** | 673,893 |  |  |
| 7-10 |  | 10,649 (3.67) | 13,714 (3.57) |
| <7 |  | 279,278 (96.33) | 370,252 (96.43) |
| **Birthweight** (kg) | 686,284 | 3.47 (3.47-3.48) | 3.49 (3.49-3.50) |
| **NNU admission** | 674,413 |  |  |
| Not admitted |  | 275,323 (94.73) | 364,827 (95.06) |
| Admitted (up to 48hrs) |  | 8,587 (2.95) | 10,354 (2.70) |
| Admitted (beyond 48hrs) |  | 6,718 (2.31) | 8,604 (2.24) |

BMI – Body Mass Index, SIMD – Scottish Index of Multiple Deprivation; NNU – Neonatal Unit

n (%) – percentage presented in columns.
