# Supplementary material for: Association of birthweight centiles and early childhood development of singleton infants born from 37 weeks of gestation in Scotland: A population-based cohort study
Source: PLoS Med. 2022 Oct 11;19(10):e1004108. doi: 10.1371/journal.pmed.1004108 (PMC9553050; doi:10.1371/journal.pmed.1004108)
Supplement: S5 Table — €—Inclusive of preterm births (gestational ages 28+0 to 43+6 weeks), n = 727,002. §–Analysis was adjusted for maternal age, BMI, parity, year of birth, gestational age at delivery, child’s sex, smoking, substance misuse in pregnancy, alcohol intake, socioeconomic status, ethnicity, diabetes, pre-eclampsia, maternal infection during pregnancy, history of stillbirth and spontaneous abortion, induction of labour, mode of delivery, use of analgesia/anaesthesia in labour, Apgar score at 5 minutes, and neonatal unit admission. ¥–Analysis was adjusted for birthweight, maternal age, parity, year of birth, child’s sex, smoking, substance misuse in pregnancy, alcohol intake, socioeconomic status, ethnicity, diabetes, pre-eclampsia, maternal infection during pregnancy, history of stillbirth and spontaneous abortion, induction of labour, mode of delivery, use of analgesia/anaesthesia in labour, Apgar score at 5 minutes, and neonatal unit admission. ₣–Analysis was adjusted for birthweight, maternal age, BMI, parity, gestational age at delivery, child’s sex, smoking, substance misuse in pregnancy, alcohol intake, socioeconomic status, ethnicity, diabetes, pre-eclampsia, maternal infection during pregnancy, history of stillbirth and spontaneous abortion, induction of labour, mode of delivery, use of analgesia/anaesthesia in labour, Apgar score at 5 minutes, and neonatal unit admission. (DOCX) [file pmed.1004108.s006.docx]

S5 Table. Predicting missingness of outcome variables for whole birth population ^€^

| **Predictor** | **Missingness of outcome variables** | | | |
| --- | --- | --- | --- | --- |
|  | *Unadjusted* | *P value* | *Adjusted* | *P value* |
| **Birthweight** ^§^ |  |  |  |  |
| <3^rd^ | 0.93 (0.92-0.95) | <0.001 | 0.95 (0.91-0.99) | 0.029 |
| 3^rd^ – 9^th^ | 0.97 (0.96-0.97) | <0.001 | 0.97 (0.95-0.99) | 0.009 |
| 10^th^ – 24^th^ | 0.99 (0.98-0.99) | <0.001 | 1.00 (0.98-1.01) | 0.933 |
| 25^th^ – 74^th^ (ref) |  |  |  |  |
| 75^th^ – 89^th^ | 1.01 (1.00-1.01) | 0.007 | 0.99 (0.98-1.01) | 0.270 |
| 90^th^ – 96^th^ | 1.01 (1.00-1.02) | 0.004 | 1.01 (0.99-1.03) | 0.335 |
| ≥97^th^ | 1.00 (0.99-1.02) | 0.374 | 0.97 (0.94-1.00) | 0.092 |
| **Gestational age (weeks)** ^¥^ |  |  |  |  |
| Preterm | 0.94 (0.93-0.95) | <0.001 | 1.00 (0.97-1.03) | 0.991 |
| Term *(ref)* |  |  |  |  |
| **Year of birth** ^₣^ |  |  |  |  |
| 2003 – 2007 | 1.11 (1.10-1.11) | <0.001 | 1.29 (1.18-1.41) | <0.001 |
| 2008 – 2011 (ref) |  |  |  |  |
| 2012 – 2015 | 0.48 (0.47-0.48) | <0.001 | 0.59 (0.58-0.60) | <0.001 |

^€^ - inclusive of preterm births (gestational ages 28^+0^ to 43^+6^ weeks), n= 727,002

§ – Analysis was adjusted for maternal age, BMI, parity, year of birth, gestational age at delivery, child’s sex, smoking, substance misuse in pregnancy, alcohol intake, socioeconomic status, ethnicity, diabetes, pre-eclampsia, maternal infection during pregnancy, history of stillbirth and spontaneous abortion, induction of labour, mode of delivery, use of analgesia/anaesthesia in labour, Apgar score at 5 minutes and neonatal unit admission.

¥ – Analysis was adjusted for birthweight, maternal age, parity, year of birth, child’s sex, smoking, substance misuse in pregnancy, alcohol intake, socioeconomic status, ethnicity, diabetes, pre-eclampsia, maternal infection during pregnancy, history of stillbirth and spontaneous abortion, induction of labour, mode of delivery, use of analgesia/anaesthesia in labour, Apgar score at 5 minutes and neonatal unit admission.

₣ – Analysis was adjusted for birthweight, maternal age, BMI, parity, gestational age at delivery, child’s sex, smoking, substance misuse in pregnancy, alcohol intake, socioeconomic status, ethnicity, diabetes, pre-eclampsia, maternal infection during pregnancy, history of stillbirth and spontaneous abortion, induction of labour, mode of delivery, use of analgesia/anaesthesia in labour, Apgar score at 5 minutes and neonatal unit admission.
