# Supplementary material for: Association of birthweight centiles and early childhood development of singleton infants born from 37 weeks of gestation in Scotland: A population-based cohort study
Source: PLoS Med. 2022 Oct 11;19(10):e1004108. doi: 10.1371/journal.pmed.1004108 (PMC9553050; doi:10.1371/journal.pmed.1004108)
Supplement: S7 Table — –n = 686,284. Analysis was adjusted for maternal age, BMI, parity, year of birth, gestational age at delivery, child’s sex, smoking, substance misuse in pregnancy, alcohol intake, socioeconomic status, ethnicity, diabetes, pre-eclampsia, maternal infection during pregnancy, history of stillbirth and spontaneous abortion, and induction of labour. (DOCX) [file pmed.1004108.s008.docx]

S7 Table. Adjusted relative risks (RR) of developmental concerns of imputed data (all infants born from 37 weeks of gestation)

|  | **Birth weight centile** | **Risk of any developmental concern** | | **Risk for each domain** | | | | | | | |
| --- | --- | --- | --- | --- | --- | --- | --- | --- | --- | --- | --- |
|  |  |  |  | **Fine motor concern** | | **Gross motor concern** | | **Communication concern** | | **Social skills concern** | |
|  |  | *RR (95% CI)* | *p value* | *RR (95% CI)* | *p value* | *RR (95% CI)* | *p value* | *RR (95% CI)* | *p value* | *RR (95% CI)* | *p value* |
| **Adjusted analysis for imputed data** ^¶^ | 25^th^ – 74^th^ (ref) |  |  |  |  |  |  |  |  |  |  |
|  | <3^rd^ | 1.37 (1.28-1.47) | <0.001 | 2.27 (1.96-2.63) | <0.001 | 2.15 (1.81-2.55) | <0.001 | 1.38 (1.28-1.48) | <0.001 | 1.57 (1.38-1.78) | <0.001 |
|  | 3^rd^ – 9^th^ | 1.15 (1.10-1.20) | <0.001 | 1.41 (1.31-1.51) | <0.001 | 1.42 (1.30-1.54) | <0.001 | 1.15 (1.10-1.21) | <0.001 | 1.26 (1.19-1.35) | <0.001 |
|  | 10^th^ – 24^th^ | 1.06 (1.03-1.09) | <0.001 | 1.20 (1.13-1.27) | <0.001 | 1.17 (1.08-1.26) | <0.001 | 1.06 (1.02-1.09) | 0.002 | 1.10 (1.05-1.16) | <0.001 |
|  | 75^th^ – 89^th^ | 0.99 (0.97-1.02) | 0.519 | 0.93 (0.86-1.00) | 0.055 | 0.93 (0.86-1.02) | 0.120 | 1.00 (0.97-1.02) | 0.909 | 0.97 (0.92-1.02) | 0.227 |
|  | 90^th^ – 96^th^ | 0.98 (0.95-1.02) | 0.319 | 0.94 (0.83-1.06) | 0.280 | 0.90 (0.78-1.04) | 0.134 | 0.99 (0.96-1.02) | 0.515 | 0.95 (0.88-1.03) | 0.226 |
|  | ≥97^th^ | 1.01 (0.95-1.06) | 0.784 | 1.02 (0.86-1.22) | 0.769 | 0.97 (0.85-1.12) | 0.698 | 1.01 (0.96-1.06) | 0.703 | 1.04 (0.94-1.16) | 0.421 |

¶ – n=686,284. Analysis was adjusted for maternal age, body mass index (BMI), parity, year of birth, gestational age at delivery, child’s sex, smoking, substance misuse in pregnancy, alcohol intake, socioeconomic status, ethnicity, diabetes, pre-eclampsia, maternal infection during pregnancy, history of stillbirth and spontaneous abortion, and induction of labour.
