# Supplementary material for: Association of birthweight centiles and early childhood development of singleton infants born from 37 weeks of gestation in Scotland: A population-based cohort study
Source: PLoS Med. 2022 Oct 11;19(10):e1004108. doi: 10.1371/journal.pmed.1004108 (PMC9553050; doi:10.1371/journal.pmed.1004108)
Supplement: S8 Table — ¥—n = 113,794, analysis was adjusted for confounders (maternal age, BMI, parity, year of birth, gestational age at delivery, child’s sex, smoking, substance misuse in pregnancy, alcohol intake, socioeconomic status, ethnicity, diabetes, pre-eclampsia, maternal infection during pregnancy, history of stillbirth and spontaneous abortion, and induction of labour) and potential mediators (mode of delivery, use of analgesia/anaesthesia in labour, Apgar score at 5 minutes, and special baby care unit admission). ₣—n = 113,794, analysis was adjusted for confounders (maternal age, BMI, parity, year of birth, gestational age at delivery, child’s sex, smoking, substance misuse in pregnancy, alcohol intake, socioeconomic status, ethnicity, diabetes, pre-eclampsia, maternal infection during pregnancy, history of stillbirth and spontaneous abortion, and induction of labour), potential mediators (mode of delivery, use of analgesia/anaesthesia in labour, Apgar score at 5 minutes, and special baby care unit admission), and child’s age at developmental assessment. (DOCX) [file pmed.1004108.s009.docx]

S8 Table. Adjusted relative risks (RR) of developmental concerns after adjusting for potential mediators and child’s age at developmental assessment (for gestational age 37^+0^ to 43^+6^).

|  | **Birth weight centile** | **Risk of any developmental concern** | | **Risk for each domain** | | | | | | | |
| --- | --- | --- | --- | --- | --- | --- | --- | --- | --- | --- | --- |
|  |  |  |  | **Fine motor concern** | | **Gross motor concern** | | **Communication concern** | | **Social skills concern** | |
|  |  | *RR (95% CI)* | *p value* | *RR (95% CI)* | *p value* | *RR (95% CI)* | *p value* | *RR (95% CI)* | *p value* | *RR (95% CI)* | *p value* |
| **Adjusted for confounders ^¥^** | 25^th^ – 74^th^ (ref) |  |  |  |  |  |  |  |  |  |  |
|  | <3^rd^ | 1.31 (1.19-1.44) | <0.001 | 1.84 (1.47-2.29) | <0.001 | 2.03 (1.61-2.55) | <0.001 | 1.28 (1.15-1.43) | <0.001 | 1.35 (1.12-1.63) | 0.002 |
|  | 3^rd^ – 9^th^ | 1.17 (1.11-1.24) | <0.001 | 1.39 (1.21-1.61) | <0.001 | 1.37 (1.17-1.60) | <0.001 | 1.17 (1.10-1.25) | <0.001 | 1.30 (1.17-1.45) | <0.001 |
|  | 10^th^ – 24^th^ | 1.08 (1.03-1.12) | 0.001 | 1.21 (1.08-1.35) | 0.001 | 1.16 (1.03-1.31) | 0.013 | 1.07 (1.02-1.12) | 0.005 | 1.17 (1.08-1.27) | <0.001 |
|  | 75^th^ – 89^th^ | 1.01 (0.97-1.06) | 0.553 | 1.06 (0.94-1.19) | 0.338 | 1.02 (0.90-1.15) | 0.740 | 1.01 (0.97-1.06) | 0.559 | 1.07 (0.99-1.17) | 0.097 |
|  | 90^th^ – 96^th^ | 1.00 (0.94-1.05) | 0.866 | 0.99 (0.84-1.17) | 0.941 | 0.88 (0.74-1.05) | 0.160 | 1.00 (0.94-1.06) | 0.968 | 1.02 (0.90-1.15) | 0.799 |
|  | ≥97^th^ | 1.02 (0.95-1.09) | 0.636 | 1.00 (0.82-1.23) | 0.997 | 1.06 (0.87-1.30) | 0.549 | 1.03 (0.95-1.11) | 0.487 | 1.03 (0.89-1.20) | 0.678 |
|  | | | | | | | | | | | |
| **Adjusted for mediators ^¶^** | 25^th^ – 74^th^ (ref) |  |  |  |  |  |  |  |  |  |  |
|  | <3^rd^ | 1.31 (1.19-1.44) | <0.001 | 1.83 (1.47-2.29) | <0.001 | 2.03 (1.61-2.55) | <0.001 | 1.28 (1.15-1.43) | <0.001 | 1.35 (1.12-1.63) | 0.002 |
|  | 3^rd^ – 9^th^ | 1.17 (1.11-1.24) | <0.001 | 1.39 (1.21-1.60) | <0.001 | 1.37 (1.17-1.60) | <0.001 | 1.17 (1.10-1.25) | <0.001 | 1.30 (1.17-1.45) | <0.001 |
|  | 10^th^ – 24^th^ | 1.08 (1.03-1.12) | 0.001 | 1.21 (1.08-1.35) | 0.001 | 1.16 (1.03-1.31) | 0.013 | 1.07 (1.02-1.12) | 0.005 | 1.17 (1.08-1.27) | <0.001 |
|  | 75^th^ – 89^th^ | 1.01 (0.97-1.06) | 0.553 | 1.06 (0.94-1.19) | 0.336 | 1.02 (0.90-1.15) | 0.739 | 1.01 (0.97-1.06) | 0.561 | 1.07 (0.99-1.17) | 0.097 |
|  | 90^th^ – 96^th^ | 1.00 (0.94-1.05) | 0.884 | 1.00 (0.85-1.18) | 0.970 | 0.88 (0.74-1.05) | 0.164 | 1.00 (0.94-1.06) | 0.990 | 1.02 (0.90-1.15) | 0.803 |
|  | ≥97^th^ | 1.02 (0.95-1.09) | 0.630 | 1.00 (0.82-1.23) | 0.993 | 1.06 (0.87-1.30) | 0.547 | 1.03 (0.95-1.11) | 0.479 | 1.03 (0.89-1.20) | 0.680 |

**^¥^** - n=113,794, analysis was adjusted for **confounders** (maternal age, body mass index (BMI), parity, year of birth, gestational age at delivery, child’s sex, smoking, substance misuse in pregnancy, alcohol intake, socioeconomic status, ethnicity, diabetes, pre-eclampsia, maternal infection during pregnancy, history of stillbirth and spontaneous abortion, and induction of labour) and **potential mediators** (mode of delivery, use of analgesia/anaesthesia in labour, Apgar score at 5 minute, and special baby care unit admission).

**^¶^** - n=113,794, analysis was adjusted for **confounders** (maternal age, body mass index (BMI), parity, year of birth, gestational age at delivery, child’s sex, smoking, substance misuse in pregnancy, alcohol intake, socioeconomic status, ethnicity, diabetes, pre-eclampsia, maternal infection during pregnancy, history of stillbirth and spontaneous abortion, and induction of labour), **potential mediators** (mode of delivery, use of analgesia/anaesthesia in labour, Apgar score at 5 minute, and special baby care unit admission and **child’s age at developmental assessment**.
