# Supplementary material for: Association of birthweight centiles and early childhood development of singleton infants born from 37 weeks of gestation in Scotland: A population-based cohort study
Source: PLoS Med. 2022 Oct 11;19(10):e1004108. doi: 10.1371/journal.pmed.1004108 (PMC9553050; doi:10.1371/journal.pmed.1004108)
Supplement: S9 Table — §–Unadjusted, CCA, n = 295,200. ¥—n = 118,325. Analysis was adjusted for confounders (maternal age, BMI, parity, year of birth, gestational age at delivery, child’s sex, smoking, substance misuse in pregnancy, alcohol intake, socioeconomic status, ethnicity, diabetes, pre-eclampsia, maternal infection during pregnancy, history of stillbirth and spontaneous abortion, and induction of labour). ¶—n = 113,794. Analysis adjusted for confounders (maternal age, BMI, parity, year of birth, gestational age at delivery, child’s sex, smoking, substance misuse in pregnancy, alcohol intake, socioeconomic status, ethnicity, diabetes, pre-eclampsia, maternal infection during pregnancy, history of stillbirth and spontaneous abortion, and induction of labour) and potential mediators (mode of delivery, use of analgesia/anaesthesia in labour, Apgar score at 5 minutes, and special baby care unit admission). (DOCX) [file pmed.1004108.s010.docx]

S9 Table. Relative risks (RR) of developmental concerns of SGA (<2500g) and LGA (>4000g) referent to birthweight between 2500g and 4000g (for gestational age 37^+0^ to 43^+6^).

|  | **Birth weight**  **(kg)** | **Risk of any developmental concern** | | **Risk for each domain** | | | | | | | |
| --- | --- | --- | --- | --- | --- | --- | --- | --- | --- | --- | --- |
|  |  |  |  | **Fine motor concern** | | **Gross motor concern** | | **Communication concern** | | **Social skills concern** | |
|  |  | *RR (95% CI)* | *p value* | *RR (95% CI)* | *p value* | *RR (95% CI)* | *p value* | *RR (95% CI)* | *p value* | *RR (95% CI)* | *p value* |
| **Univariate analysis** ^§^ | 2.5 to 4.0 (ref) |  |  |  |  |  |  |  |  |  |  |
|  | <2.5 | 1.47 (1.4-1.54) | <0.001 | 2.36 (2.13-2.61) | <0.001 | 2.17 (1.93-2.43) | <0.001 | 1.45 (1.38-1.53) | <0.001 | 1.75 (1.60-1.92) | <0.001 |
|  | >4.0 | 1.00 (0.97-1.03) | 0.976 | 0.89 (0.83-0.96) | 0.002 | 0.88 (0.82-0.96) | 0.002 | 1.01 (0.99-1.04) | 0.335 | 0.96 (0.91-1.01) | 0.141 |
|  | | | | | | | | | | | |
| **Adjusted for confounders**^¥^ | 2.5 to 4.0 (ref) |  |  |  |  |  |  |  |  |  |  |
|  | <2.5 | 1.32 (1.21-1.43) | <0.001 | 1.61 (1.32-1.96) | <0.001 | 1.77 (1.43-2.17) | <0.001 | 1.31 (1.19-1.43) | <0.001 | 1.41 (1.21-1.65) | <0.001 |
|  | >4.0 | 1.01 (0.97-1.05) | 0.684 | 1.04 (0.92-1.16) | 0.538 | 1.02 (0.90-1.15) | 0.769 | 1.01 (0.96-1.05) | 0.806 | 1.07 (0.98-1.16) | 0.137 |
|  | | | | | | | | | | | |
| **Adjusted for mediators** ^¶^ | 2.5 to 4.0 (ref) |  |  |  |  |  |  |  |  |  |  |
|  | <2.5 | 1.28 (1.17-1.39) | <0.001 | 1.44 (1.17-1.78) | <0.001 | 1.58 (1.27-1.97) | <0.001 | 1.26 (1.14-1.38) | <0.001 | 1.35 (1.15-1.59) | <0.001 |
|  | >4.0 | 1.00 (0.96-1.05) | 0.873 | 1.02 (0.90-1.15) | 0.771 | 1.00 (0.88-1.13) | 0.945 | 1.00 (0.96-1.05) | 0.966 | 1.04 (0.95-1.14) | 0.360 |

§ – Unadjusted, complete case analysis (CCA), n=295,200.

¥ - n=118,325. Analysis was adjusted for **confounders** (maternal age, body mass index (BMI), parity, year of birth, gestational age at delivery, child’s sex, smoking, substance misuse in pregnancy, alcohol intake, socioeconomic status, ethnicity, diabetes, pre-eclampsia, maternal infection during pregnancy, history of stillbirth and spontaneous abortion, and induction of labour).

¶ - n=113,794, Analysis adjusted for **confounders** (maternal age, body mass index (BMI), parity, year of birth, gestational age at delivery, child’s sex, smoking, substance misuse in pregnancy, alcohol intake, socioeconomic status, ethnicity, diabetes, pre-eclampsia, maternal infection during pregnancy, history of stillbirth and spontaneous abortion, and induction of labour) and **potential mediators** (mode of delivery, use of analgesia/anaesthesia in labour, Apgar score at 5 minute, special baby care unit admission).
