# Supplementary material for: Association of birthweight centiles and early childhood development of singleton infants born from 37 weeks of gestation in Scotland: A population-based cohort study
Source: PLoS Med. 2022 Oct 11;19(10):e1004108. doi: 10.1371/journal.pmed.1004108 (PMC9553050; doi:10.1371/journal.pmed.1004108)
Supplement: S10 Table — §–Unadjusted, CCA, n = 295,200. ¥—n = 118,325. Analysis was adjusted for confounders (maternal age, BMI, parity, year of birth, gestational age at delivery, child’s sex, smoking, substance misuse in pregnancy, alcohol intake, socioeconomic status, ethnicity, diabetes, pre-eclampsia, maternal infection during pregnancy, history of stillbirth and spontaneous abortion, and induction of labour). ¶—n = 113,794. Analysis adjusted for confounders (maternal age, BMI, parity, year of birth, gestational age at delivery, child’s sex, smoking, substance misuse in pregnancy, alcohol intake, socioeconomic status, ethnicity, diabetes, pre-eclampsia, maternal infection during pregnancy, history of stillbirth and spontaneous abortion, and induction of labour) and potential mediators (mode of delivery, use of analgesia/anaesthesia in labour, Apgar score at 5 minutes, and special baby care unit admission). (DOCX) [file pmed.1004108.s011.docx]

S10 Table. Relative risks (RR) of developmental concerns using more granular absolute birthweight categories (gestational age 37^+0^ to 43^+6^).

|  | **Birth weight**  **(kg)** | **Risk of any developmental concern** | | **Risk for each domain** | | | | | | | |
| --- | --- | --- | --- | --- | --- | --- | --- | --- | --- | --- | --- |
|  |  |  |  | **Fine motor concern** | | **Gross motor concern** | | **Communication concern** | | **Social skills concern** | |
|  |  | *RR (95% CI)* | *p value* | *RR (95% CI)* | *p value* | *RR (95% CI)* | *p value* | *RR (95% CI)* | *p value* | *RR (95% CI)* | *p value* |
| **Univariate analysis** ^§^ | 3.0 to 4.0 (ref) |  |  |  |  |  |  |  |  |  |  |
|  | <2.5 | 1.51 (1.44-1.58) | <0.001 | 2.53 (2.28-2.80) | <0.001 | 2.32 (2.06-2.61) | <0.001 | 1.49 (1.41-1.57) | <0.001 | 1.86 (1.69-2.04) | <0.001 |
|  | 2.5 to 2.99 | 1.17 (1.14-1.20) | <0.001 | 1.46 (1.37-1.55) | <0.001 | 1.43 (1.34-1.53) | <0.001 | 1.15 (1.12-1.18) | <0.001 | 1.36 (1.30-1.43) | <0.001 |
|  | >4.0 | 1.03 (1.00-1.06) | 0.047 | 0.96 (0.89-1.03) | 0.257 | 0.95 (0.87-1.02) | 0.170 | 1.04 (1.01-1.07) | 0.008 | 1.02 (0.96-1.08) | 0.579 |
|  | | | | | | | | | | | |
| **Adjusted for confounders**^¥^ | 3.0 to 4.0 (ref) |  |  |  |  |  |  |  |  |  |  |
|  | <2.5 | 1.37 (1.26-1.50) | <0.001 | 1.78 (1.45-2.19) | <0.001 | 1.95 (1.57-2.41) | <0.001 | 1.36 (1.24-1.50) | <0.001 | 1.52 (1.29-1.78) | <0.001 |
|  | 2.5 to 2.99 | 1.12 (1.08-1.17) | <0.001 | 1.29 (1.15-1.44) | <0.001 | 1.28 (1.13-1.44) | <0.001 | 1.12 (1.07-1.18) | <0.001 | 1.20 (1.11-1.31) | <0.001 |
|  | >4.0 | 1.02 (0.98-1.06) | 0.401 | 1.06 (0.94-1.19) | 0.321 | 1.04 (0.92-1.18) | 0.521 | 1.02 (0.97-1.06) | 0.517 | 1.08 (1.00-1.18) | 0.064 |
|  | | | | | | | | | | | |
| **Adjusted for mediators** ^¶^ | 3.0 to 4.0 (ref) |  |  |  |  |  |  |  |  |  |  |
|  | <2.5 | 1.33 (1.22-1.45) | <0.001 | 1.60 (1.29-1.98) | <0.001 | 1.74 (1.39-2.18) | <0.001 | 1.31 (1.19-1.45) | <0.001 | 1.45 (1.23-1.72) | <0.001 |
|  | 2.5 to 2.99 | 1.12 (1.07-1.17) | <0.001 | 1.29 (1.16-1.45) | <0.001 | 1.27 (1.13-1.44) | <0.001 | 1.12 (1.07-1.18) | <0.001 | 1.20 (1.10-1.31) | <0.001 |
|  | >4.0 | 1.01 (0.97-1.06) | 0.564 | 1.04 (0.92-1.17) | 0.504 | 1.02 (0.90-1.15) | 0.787 | 1.01 (0.96-1.06) | 0.666 | 1.06 (0.97-1.15) | 0.204 |

§ – Unadjusted, complete case analysis (CCA), n=295,200.

¥ - n=118,325. Analysis was adjusted for **confounders** (maternal age, body mass index (BMI), parity, year of birth, gestational age at delivery, child’s sex, smoking, substance misuse in pregnancy, alcohol intake, socioeconomic status, ethnicity, diabetes, pre-eclampsia, maternal infection during pregnancy, history of stillbirth and spontaneous abortion, and induction of labour).

¶ - n=113,794, Analysis adjusted for **confounders** (maternal age, body mass index (BMI), parity, year of birth, gestational age at delivery, child’s sex, smoking, substance misuse in pregnancy, alcohol intake, socioeconomic status, ethnicity, diabetes, pre-eclampsia, maternal infection during pregnancy, history of stillbirth and spontaneous abortion, and induction of labour) and **potential mediators** (mode of delivery, use of analgesia/anaesthesia in labour, Apgar score at 5 minute, special baby care unit admission).
