# Supplementary material for: Association of birthweight centiles and early childhood development of singleton infants born from 37 weeks of gestation in Scotland: A population-based cohort study
Source: PLoS Med. 2022 Oct 11;19(10):e1004108. doi: 10.1371/journal.pmed.1004108 (PMC9553050; doi:10.1371/journal.pmed.1004108)
Supplement: S11 Table — §: n = 287,414. ¥: n = 115,314. Adjusted for maternal age, BMI, parity, year of birth, gestational age at delivery, child’s sex, smoking, illicit drug use in pregnancy, alcohol intake, socioeconomic status, ethnicity, diabetes, pre-eclampsia, maternal infection during pregnancy, history of stillbirth and spontaneous abortion, and induction of labour. (DOCX) [file pmed.1004108.s012.docx]

S11 Table. The proportion of children by birthweight centiles with developmental concern in each domain (gestational age 37^+0^ to 41^+6^)

|  | **Birth weight centile** | **Frequency, n** | **Risk of any developmental concern** | | **Risk for each domain** | | | | | | | |
| --- | --- | --- | --- | --- | --- | --- | --- | --- | --- | --- | --- | --- |
|  |  |  |  |  | **Fine motor concern** | | **Gross motor concern** | | **Communication concern** | | **Social skills concern** | |
|  |  |  | *No* | *Yes* | *No* | *Yes* | *No* | *Yes* | *No* | *Yes* | *No* | *Yes* |
| **Unadjusted complete follow-up data** ^§^ | <3^rd^ | 5,260 | 4,160 (79.09) | 1,100 (20.91) | 4,958 (94.26) | 302 (5.74) | 5,029 (95.61) | 231 (4.39) | 4,310 (81.94) | 950 (18.06) | 4,939 (93.90) | 321 (6.10) |
|  | 3^rd^ – 9^th^ | 19,037 | 15,749 (82.73) | 3,288 (17.27) | 18,358 (96.43) | 679 (3.57) | 18,503 (97.19) | 534 (2.81) | 16,163 (84.9) | 2,874 (15.10) | 18,071 (94.93) | 966 (5.07) |
|  | 10^th^ – 24^th^ | 42,159 | 35,615 (84.48) | 6,544 (15.52) | 40,914 (97.05) | 1,245 (2.95) | 41,147 (97.6) | 1,012 (2.40) | 36,496 (86.57) | 5,663 (13.43) | 40,332 (95.67) | 1,827 (4.33) |
|  | 25^th^ – 74^th^ | 143,936 | 124,241 (86.32) | 19,695 (13.68) | 140,825 (97.84) | 3,111 (2.16) | 141,277 (98.15) | 2,659 (1.85) | 126,751 (88.06) | 17,185 (11.94) | 138,942 (96.53) | 4,994 (3.47) |
|  | 75^th^ – 89^th^ | 44,069 | 38,187 (86.65) | 5,882 (13.35) | 43,194 (98.01) | 875 (1.99) | 43,282 (98.21) | 787 (1.79) | 38,900 (88.27) | 5,169 (11.73) | 42,643 (96.76) | 1,426 (3.24) |
|  | 90^th^ – 96^th^ | 20,689 | 17,966 (86.84) | 2,723 (13.16) | 20,298 (98.11) | 391 (1.89) | 20,335 (98.29) | 354 (1.71) | 18,312 (88.51) | 2,377 (11.49) | 20,025 (96.79) | 664 (3.21) |
|  | ≥97^th^ | 12,264 | 10,591 (86.36) | 1,673 (13.64) | 12,001 (97.86) | 263 (2.14) | 12,042 (98.19) | 222 (1.81) | 10,785 (87.94) | 1,479 (12.06) | 11,851 (96.63) | 413 (3.37) |
|  | | | | | | | | | | | | |
| **Adjusted complete follow-up data** ^¥^ | <3^rd^ | 1,655 | 1,298 (78.43) | 357 (21.57) | 1,570 (94.86) | 85 (5.14) | 1,572 (94.98) | 83 (5.02) | 1,357 (81.99) | 298 (18.01) | 1,543 (93.23) | 112 (6.77) |
|  | 3^rd^ – 9^th^ | 6,755 | 5,550 (82.16) | 1,205 (17.84) | 6,530 (96.67) | 225 (3.33) | 6,560 (97.11) | 195 (2.89) | 5,724 (84.74) | 1,031 (15.26) | 6,365 (94.23) | 390 (5.77) |
|  | 10^th^ – 24^th^ | 16,022 | 13,574 (84.72) | 2,448 (15.28) | 15,604 (97.39) | 418 (2.61) | 15,659 (97.73) | 363 (2.27) | 13,933 (86.96) | 2,089 (13.04) | 15,274 (95.33) | 748 (4.67) |
|  | 25^th^ – 74^th^ | 58,483 | 50,620 (86.56) | 7,863 (13.44) | 57,327 (98.02) | 1,156 (1.98) | 57,404 (98.16) | 1,079 (1.84) | 51,708 (88.42) | 6,775 (11.58) | 56,380 (96.40) | 2,103 (3.60) |
|  | 75^th^ – 89^th^ | 18,532 | 16,065 (86.69) | 2,467 (13.31) | 18,164 (98.01) | 368 (1.99) | 18,194 (98.18) | 338 (1.82) | 16,395 (88.47) | 2,137 (11.53) | 17,858 (96.36) | 674 (3.64) |
|  | 90^th^ – 96^th^ | 8,698 | 7,565 (86.97) | 1,133 (13.03) | 8,536 (98.14) | 162 (1.86) | 8,559 (98.40) | 139 (1.60) | 7,715 (88.70) | 983 (11.30) | 8,400 (96.57) | 298 (3.43) |
|  | ≥97^th^ | 5,169 | 4,448 (86.05) | 721 (13.95) | 5,065 (97.99) | 104 (2.01) | 5,059 (97.87) | 110 (2.13) | 4,536 (87.75) | 633 (12.25) | 4,986 (96.46) | 183 (3.54) |

§: n=287,414

¥: n=115,314. Adjusted for maternal age, body mass index (BMI), parity, year of birth, gestational age at delivery, child’s sex, smoking, illicit drug use in pregnancy, alcohol intake, socioeconomic status, ethnicity, diabetes, pre-eclampsia, maternal infection during pregnancy, history of stillbirth and spontaneous abortion, and induction of labour.

Data presented as n (%).
