# Supplementary material for: Association of birthweight centiles and early childhood development of singleton infants born from 37 weeks of gestation in Scotland: A population-based cohort study
Source: PLoS Med. 2022 Oct 11;19(10):e1004108. doi: 10.1371/journal.pmed.1004108 (PMC9553050; doi:10.1371/journal.pmed.1004108)
Supplement: S12 Table — §–Unadjusted, CCA, n = 287,414. ¥—n = 115,314. Analysis was adjusted for confounders (maternal age, BMI, parity, year of birth, gestational age at delivery, child’s sex, smoking, substance misuse in pregnancy, alcohol intake, socioeconomic status, ethnicity, diabetes, pre-eclampsia, maternal infection during pregnancy, history of stillbirth and spontaneous abortion, and induction of labour). ¶—n = 110,877. Analysis adjusted for confounders (maternal age, BMI, parity, year of birth, gestational age at delivery, child’s sex, smoking, substance misuse in pregnancy, alcohol intake, socioeconomic status, ethnicity, diabetes, pre-eclampsia, maternal infection during pregnancy, history of stillbirth and spontaneous abortion, and induction of labour) and potential mediators (mode of delivery, use of analgesia/anaesthesia in labour, Apgar score at 5 minutes, and special baby care unit admission). (DOCX) [file pmed.1004108.s013.docx]

S12 Table. Relative risks (RR) of developmental concerns for gestational age 37^+0^ to 41^+6^

|  | **Birth weight centile** | **Risk of any developmental concern** | | **Risk for each domain** | | | | | | | |
| --- | --- | --- | --- | --- | --- | --- | --- | --- | --- | --- | --- |
|  |  |  |  | **Fine motor concern** | | **Gross motor concern** | | **Communication concern** | | **Social skills concern** | |
|  |  | *RR (95% CI)* | *p value* | *RR (95% CI)* | *p value* | *RR (95% CI)* | *p value* | *RR (95% CI)* | *p value* | *RR (95% CI)* | *p value* |
| **Unadjusted analysis** ^§^ | 25^th^ – 74^th^ (ref) |  |  |  |  |  |  |  |  |  |  |
|  | <3^rd^ | 1.53 (1.45-1.61) | <0.001 | 2.66 (2.37-2.98) | <0.001 | 2.38 (2.08-2.71) | <0.001 | 1.51 (1.43-1.61) | <0.001 | 1.76 (1.58-1.96) | <0.001 |
|  | 3^rd^ – 9^th^ | 1.26 (1.22-1.31) | <0.001 | 1.65 (1.52-1.79) | <0.001 | 1.52 (1.39-1.66) | <0.001 | 1.26 (1.22-1.31) | <0.001 | 1.46 (1.37-1.56) | <0.001 |
|  | 10^th^ – 24^th^ | 1.13 (1.11-1.16) | <0.001 | 1.37 (1.28-1.46) | <0.001 | 1.30 (1.21-1.40) | <0.001 | 1.13 (1.09-1.16) | <0.001 | 1.25 (1.18-1.32) | <0.001 |
|  | 75^th^ – 89^th^ | 0.98 (0.95-1.00) | 0.073 | 0.92 (0.85-0.99) | 0.025 | 0.97 (0.89-1.05) | 0.401 | 0.98 (0.95-1.01) | 0.236 | 0.93 (0.88-0.99) | 0.018 |
|  | 90^th^ – 96^th^ | 0.96 (0.93-1.00) | 0.042 | 0.87 (0.79-0.97) | 0.012 | 0.93 (0.83-1.03) | 0.175 | 0.96 (0.92-1.00) | 0.063 | 0.93 (0.85-1.00) | 0.055 |
|  | ≥97^th^ | 1.00 (0.95-1.04) | 0.898 | 0.99 (0.88-1.12) | 0.902 | 0.98 (0.86-1.12) | 0.769 | 1.01 (0.96-1.06) | 0.695 | 0.97 (0.88-1.07) | 0.557 |
|  | | | | | | | | | | | |
| **Adjusted for confounders**^¥^ | 25^th^ – 74^th^ (ref) |  |  |  |  |  |  |  |  |  |  |
|  | <3^rd^ | 1.37 (1.25-1.50) | <0.001 | 2.05 (1.65-2.55) | <0.001 | 2.35 (1.88-2.93) | <0.001 | 1.34 (1.21-1.49) | <0.001 | 1.43 (1.19-1.72) | <0.001 |
|  | 3^rd^ – 9^th^ | 1.18 (1.12-1.25) | <0.001 | 1.44 (1.25-1.65) | <0.001 | 1.42 (1.22-1.66) | <0.001 | 1.18 (1.11-1.26) | <0.001 | 1.31 (1.18-1.46) | <0.001 |
|  | 10^th^ – 24^th^ | 1.07 (1.03-1.11) | 0.002 | 1.21 (1.08-1.35) | 0.001 | 1.17 (1.04-1.32) | 0.010 | 1.06 (1.02-1.11) | 0.008 | 1.16 (1.07-1.26) | <0.001 |
|  | 75^th^ – 89^th^ | 1.01 (0.97-1.05) | 0.721 | 1.04 (0.92-1.17) | 0.523 | 0.99 (0.88-1.12) | 0.931 | 1.01 (0.96-1.06) | 0.683 | 1.07 (0.98-1.16) | 0.124 |
|  | 90^th^ – 96^th^ | 0.99 (0.94-1.05) | 0.783 | 0.98 (0.83-1.16) | 0.818 | 0.87 (0.73-1.03) | 0.109 | 0.99 (0.93-1.06) | 0.858 | 1.03 (0.91-1.16) | 0.623 |
|  | ≥97^th^ | 1.05 (0.98-1.13) | 0.176 | 1.05 (0.86-1.28) | 0.644 | 1.12 (0.92-1.36) | 0.273 | 1.06 (0.99-1.15) | 0.111 | 1.07 (0.92-1.24) | 0.367 |
|  | | | | | | | | | | | |
| **Adjusted for mediators**^¶^ | 25^th^ – 74^th^ (ref) |  |  |  |  |  |  |  |  |  |  |
|  | <3^rd^ | 1.31 (1.19-1.44) | <0.001 | 1.83 (1.46-2.29) | <0.001 | 2.04 (1.61-2.57) | <0.001 | 1.28 (1.15-1.43) | <0.001 | 1.35 (1.12-1.64) | 0.002 |
|  | 3^rd^ – 9^th^ | 1.18 (1.11-1.24) | <0.001 | 1.40 (1.21-1.62) | <0.001 | 1.37 (1.17-1.61) | <0.001 | 1.18 (1.10-1.25) | <0.001 | 1.31 (1.17-1.46) | <0.001 |
|  | 10^th^ – 24^th^ | 1.07 (1.03-1.12) | 0.001 | 1.21 (1.08-1.35) | 0.001 | 1.17 (1.04-1.32) | 0.012 | 1.07 (1.02-1.12) | 0.007 | 1.16 (1.07-1.26) | 0.001 |
|  | 75^th^ – 89^th^ | 1.01 (0.97-1.05) | 0.718 | 1.04 (0.93-1.17) | 0.500 | 1.00 (0.88-1.13) | 0.963 | 1.01 (0.96-1.05) | 0.758 | 1.06 (0.97-1.16) | 0.176 |
|  | 90^th^ – 96^th^ | 0.99 (0.94-1.05) | 0.774 | 0.98 (0.82-1.15) | 0.770 | 0.85 (0.71-1.02) | 0.077 | 0.99 (0.93-1.06) | 0.847 | 1.00 (0.89-1.13) | 0.953 |
|  | ≥97^th^ | 1.03 (0.95-1.10) | 0.481 | 0.98 (0.80-1.21) | 0.874 | 1.05 (0.86-1.28) | 0.656 | 1.04 (0.96-1.12) | 0.322 | 1.03 (0.89-1.20) | 0.669 |

§ – Unadjusted, complete case analysis (CCA), n=287,414.

¥ - n=115,314. Analysis was adjusted for **confounders** (maternal age, body mass index (BMI), parity, year of birth, gestational age at delivery, child’s sex, smoking, substance misuse in pregnancy, alcohol intake, socioeconomic status, ethnicity, diabetes, pre-eclampsia, maternal infection during pregnancy, history of stillbirth and spontaneous abortion, and induction of labour).

¶ - n=110,877, Analysis adjusted for **confounders** (maternal age, body mass index (BMI), parity, year of birth, gestational age at delivery, child’s sex, smoking, substance misuse in pregnancy, alcohol intake, socioeconomic status, ethnicity, diabetes, pre-eclampsia, maternal infection during pregnancy, history of stillbirth and spontaneous abortion, and induction of labour) and **potential mediators** (mode of delivery, use of analgesia/anaesthesia in labour, Apgar score at 5 minute, special baby care unit admission).
