# Supplementary material for: Association of birthweight centiles and early childhood development of singleton infants born from 37 weeks of gestation in Scotland: A population-based cohort study
Source: PLoS Med. 2022 Oct 11;19(10):e1004108. doi: 10.1371/journal.pmed.1004108 (PMC9553050; doi:10.1371/journal.pmed.1004108)
Supplement: S13 Table — §–Unadjusted, CCA, n = 309,193. ¥—Adjusted CCA, n = 122,263. Analysis was adjusted for maternal age, BMI, parity, year of birth, gestational age at delivery, child’s sex, smoking, substance misuse in pregnancy, alcohol intake, socioeconomic status, ethnicity, diabetes, pre-eclampsia, maternal infection during pregnancy, history of stillbirth and spontaneous abortion, and induction of labour. ¶–Adjusted analysis, imputed data, n = 727,002. Analysis was adjusted for same covariates as in CCA. (DOCX) [file pmed.1004108.s014.docx]

S13 Table. Relative risks (RR) of developmental concerns for whole birth population (infants born from 28^+0^ to 43^+6^)

|  | **Birth weight centile** | **Risk of any developmental concern** | | **Risk for each domain** | | | | | | | |
| --- | --- | --- | --- | --- | --- | --- | --- | --- | --- | --- | --- |
|  |  |  |  | **Fine motor concern** | | **Gross motor concern** | | **Communication concern** | | **Social skills concern** | |
|  |  | *RR (95% CI)* | *p value* | *RR (95% CI)* | *p value* | *RR (95% CI)* | *p value* | *RR (95% CI)* | *p value* | *RR (95% CI)* | *p value* |
| **Unadjusted analysis** ^§^ | 25^th^ – 74^th^ (ref) |  |  |  |  |  |  |  |  |  |  |
|  | <3^rd^ | 1.54 (1.46-1.61) | <0.001 | 2.67 (2.41-2.95) | <0.001 | 2.47 (2.21-2.76) | <0.001 | 1.52 (1.45-1.61) | <0.001 | 1.80 (1.64-1.99) | <0.001 |
|  | 3^rd^ – 9^th^ | 1.26 (1.22-1.30) | <0.001 | 1.65 (1.53-1.78) | <0.001 | 1.51 (1.39-1.64) | <0.001 | 1.26 (1.22-1.31) | <0.001 | 1.46 (1.37-1.55) | <0.001 |
|  | 10^th^ – 24^th^ | 1.13 (1.10-1.15) | <0.001 | 1.34 (1.26-1.42) | <0.001 | 1.28 (1.20-1.36) | <0.001 | 1.12 (1.09-1.15) | <0.001 | 1.23 (1.17-1.29) | <0.001 |
|  | 75^th^ – 89^th^ | 0.96 (0.94-0.99) | 0.005 | 0.91 (0.85-0.97) | 0.007 | 0.95 (0.88-1.02) | 0.179 | 0.97 (0.94-0.99) | 0.018 | 0.93 (0.88-0.98) | 0.012 |
|  | 90^th^ – 96^th^ | 0.97 (0.93-1.00) | 0.073 | 0.92 (0.84-1.01) | 0.092 | 0.95 (0.88-1.05) | 0.353 | 0.97 (0.93-1.01) | 0.125 | 0.94 (0.87-1.01) | 0.115 |
|  | ≥97^th^ | 1.00 (0.96-1.05) | 0.927 | 0.97 (0.86-1.10) | 0.677 | 0.97 (0.85-1.10) | 0.658 | 1.01 (0.96-1.06) | 0.654 | 0.96 (0.87-1.05) | 0.380 |
|  | | | | | | | | | | | |
| **Adjusted analysis** ^¥^ | 25^th^ – 74^th^ (ref) |  |  |  |  |  |  |  |  |  |  |
|  | <3^rd^ | 1.36 (1.25-1.48) | <0.001 | 2.07 (1.71-2.52) | <0.001 | 2.36 (1.93-2.88) | <0.001 | 1.35 (1.23-1.49) | <0.001 | 1.45 (1.22-1.71) | <0.001 |
|  | 3^rd^ – 9^th^ | 1.18 (1.12-1.24) | <0.001 | 1.40 (1.23-1.60) | <0.001 | 1.32 (1.14-1.53) | <0.001 | 1.19 (1.12-1.26) | <0.001 | 1.31 (1.19-1.45) | <0.001 |
|  | 10^th^ – 24^th^ | 1.07 (1.02-1.11) | 0.002 | 1.20 (1.08-1.33) | 0.001 | 1.18 (1.06-1.32) | 0.003 | 1.06 (1.01-1.11) | 0.010 | 1.15 (1.06-1.24) | 0.001 |
|  | 75^th^ – 89^th^ | 1.01 (0.97-1.05) | 0.655 | 1.04 (0.93-1.16) | 0.539 | 1.01 (0.90-1.14) | 0.867 | 1.01 (0.97-1.06) | 0.659 | 1.08 (0.99-1.17) | 0.69 |
|  | 90^th^ – 96^th^ | 1.00 (0.94-1.05) | 0.917 | 1.00 (0.85-1.17) | 0.983 | 0.92 (0.77-1.08) | 0.301 | 1.00 (0.94-1.06) | 0.962 | 1.02 (0.91-1.15) | 0.741 |
|  | ≥97^th^ | 1.05 (0.98-1.13) | 0.151 | 1.06 (0.87-1.29) | 0.551 | 1.17 (0.96-1.41) | 0.119 | 1.05 (0.98-1.14) | 0.189 | 1.06 (0.92-1.23) | 0.426 |
|  | | | | | | | | | | | |
| **Adjusted analysis**  **(imputed)** ^¶^ | 25^th^ – 74^th^ (ref) |  |  |  |  |  |  |  |  |  |  |
|  | <3^rd^ | 1.38 (1.29-1.48) | <0.001 | 2.27 (1.98-2.60) | <0.001 | 2.16 (1.85-2.52) | <0.001 | 1.39 (1.30-1.48) | <0.001 | 1.59 (1.40-1.81) | <0.001 |
|  | 3^rd^ – 9^th^ | 1.16 (1.11-1.21) | <0.001 | 1.44 (1.34-1.54) | <0.001 | 1.45 (1.34-1.57) | <0.001 | 1.17 (1.11-1.22) | <0.001 | 1.28 (1.20-1.36) | <0.001 |
|  | 10^th^ – 24^th^ | 1.06 (1.03-1.09) | <0.001 | 1.20 (1.14-1.26) | <0.001 | 1.17 (1.09-1.25) | <0.001 | 1.06 (1.03-1.09) | 0.001 | 1.10 (1.05-1.15) | <0.001 |
|  | 75^th^ – 89^th^ | 0.99 (0.96-1.01) | 0.284 | 0.93 (0.86-1.01) | 0.086 | 0.94 (0.87-1.01) | 0.195 | 0.99 (0.97-1.02) | 0.577 | 0.96 (0.91-1.01) | 0.148 |
|  | 90^th^ – 96^th^ | 0.98 (0.95-1.01) | 0.211 | 0.93 (0.83-1.04) | 0.175 | 0.90 (0.79-1.01) | 0.076 | 0.99 (0.96-1.02) | 0.368 | 0.94 (0.87-1.01) | 0.101 |
|  | ≥97^th^ | 1.00 (0.95-1.06) | 0.853 | 1.02 (0.87-1.19) | 0.839 | 0.97 (0.85-1.10) | 0.609 | 1.01 (0.96-1.06) | 0.766 | 1.04 (0.94-1.15) | 0.424 |

§ – Unadjusted, complete case analysis (CCA), n= 314,161.

¥ - Adjusted CCA, n=121,681. Analysis was adjusted for maternal age, body mass index (BMI), parity, year of birth, gestational age at delivery, child’s sex, smoking, substance misuse in pregnancy, alcohol intake, socioeconomic status, ethnicity, diabetes, pre-eclampsia, maternal infection during pregnancy, history of stillbirth and spontaneous abortion, and induction of labour.

¶ – Adjusted analysis, imputed data, n=727,002. Analysis was adjusted for same covariates as in CCA.
