# Supplementary material for: Association of birthweight centiles and early childhood development of singleton infants born from 37 weeks of gestation in Scotland: A population-based cohort study
Source: PLoS Med. 2022 Oct 11;19(10):e1004108. doi: 10.1371/journal.pmed.1004108 (PMC9553050; doi:10.1371/journal.pmed.1004108)
Supplement: S14 Table — §–Unadjusted, n = 147,789. ¥—Adjusted, n = 59,977. Analysis was adjusted for maternal age, BMI, parity, year of birth, gestational age at delivery, child’s sex, smoking, substance misuse in pregnancy, alcohol intake, socioeconomic status, ethnicity, diabetes, pre-eclampsia, maternal infection during pregnancy, history of stillbirth and spontaneous abortion, and induction of labour. (DOCX) [file pmed.1004108.s015.docx]

S14 Table. Check for variance in outcome domains within 25^th^ and 74^th^ birthweight centiles (for gestational age 37^+0^ to 43^+6^).

|  | **Birth weight centile** | **Risk of any developmental concern** | | **Risk for each domain** | | | | | | | |
| --- | --- | --- | --- | --- | --- | --- | --- | --- | --- | --- | --- |
|  |  |  |  | **Fine motor concern** | | **Gross motor concern** | | **Communication concern** | | **Social skills concern** | |
|  |  | *RR (95% CI)* | *p value* | *RR (95% CI)* | *p value* | *RR (95% CI)* | *p value* | *RR (95% CI)* | *p value* | *RR (95% CI)* | *p value* |
| **Unadjusted analysis** ^§^ | 45^th^ – 54^th^ (ref) |  |  |  |  |  |  |  |  |  |  |
|  | 25^th^ – 34^th^ | 1.04 (1.00-1.09) | 0.041 | 1.13 (1.01-1.25) | 0.025 | 0.98 (0.88-1.10) | 0.784 | 1.04 (1.00-1.09) | 0.062 | 1.10 (1.01-1.20) | 0.028 |
|  | 35^th^ – 44^th^ | 1.02 (0.98-1.06) | 0.297 | 1.00 (0.90-1.11) | 0.974 | 0.95 (0.84-1.06) | 0.359 | 1.02 (0.98-1.07) | 0.291 | 1.09 (1.01-1.19) | 0.036 |
|  | 55^th^ –64^th^ | 0.94 (0.90-0.98) | 0.002 | 0.92 (0.82-1.03) | 0.137 | 0.86 (0.77-0.97) | 0.016 | 0.93 (0.89-0.98) | 0.003 | 0.94 (0.87-1.03) | 0.197 |
|  | 65^th^ – 74^th^ | 0.95 (0.91-0.99) | 0.015 | 0.93 (0.83-1.04) | 0.207 | 0.92 (0.82-1.04) | 0.171 | 0.94 (0.90-0.99) | 0.010 | 0.95 (0.87-1.04) | 0.284 |
|  | | | | | | | | | | | |
| **Adjusted analysis** ^¥^ | 45^th^ – 54^th^  (ref) |  |  |  |  |  |  |  |  |  |  |
|  | 25^th^ – 34^th^ | 1.01 (0.95-1.07) | 0.829 | 1.12 (0.94-1.33) | 0.219 | 1.02 (0.85-1.22) | 0.855 | 1.01 (0.94-1.08) | 0.768 | 0.98 (0.86-1.12) | 0.807 |
|  | 35^th^ – 44^th^ | 1.00 (0.94-1.06) | 0.978 | 0.99 (0.83-1.19) | 0.920 | 1.04 (0.87-1.25) | 0.673 | 1.01 (0.94-1.08) | 0.795 | 1.03 (0.91-1.18) | 0.605 |
|  | 55^th^ –64^th^ | 0.97 (0.91-1.03) | 0.365 | 0.96 (0.80-1.15) | 0.631 | 0.87 (0.72-1.05) | 0.152 | 0.98 (0.91-1.04) | 0.481 | 0.96 (0.84-1.10) | 0.574 |
|  | 65^th^ – 74^th^ | 0.96 (0.90-1.02) | 0.193 | 1.10 (0.92-1.31) | 0.291 | 1.02 (0.85-1.22) | 0.841 | 0.96 (0.9o-1.03) | 0.243 | 0.99 (0.87-1.12) | 0.836 |

§ – Unadjusted, n=147,789.

¥ - Adjusted, n=59,977. Analysis was adjusted for maternal age, body mass index (BMI), parity, year of birth, gestational age at delivery, child’s sex, smoking, substance misuse in pregnancy, alcohol intake, socioeconomic status, ethnicity, diabetes, pre-eclampsia, maternal infection during pregnancy, history of stillbirth and spontaneous abortion, and induction of labour.
