# Supplementary material for: Association of birthweight centiles and early childhood development of singleton infants born from 37 weeks of gestation in Scotland: A population-based cohort study
Source: PLoS Med. 2022 Oct 11;19(10):e1004108. doi: 10.1371/journal.pmed.1004108 (PMC9553050; doi:10.1371/journal.pmed.1004108)
Supplement: S15 Table — ¥—Adjustment for maternal age, BMI, parity, year of birth, gestational age at delivery, child’s sex, smoking, substance misuse in pregnancy, alcohol intake, socioeconomic status [deprivation index], ethnicity, diabetes, pre-eclampsia, maternal infection during pregnancy, history of stillbirth and spontaneous abortion, and induction of labour, referent to birthweight between 25th and 74th centile. Result presented as percent (95% CI). (DOCX) [file pmed.1004108.s016.docx]

S15 Table. Population attributable fractions (PAFs) of infants born from 37 weeks gestational age within the whole birth population (28^+0^ to 43^+6^ weeks)

|  | **Birth weight centile** | **Risk of any developmental concern** | **PAF for each domain** | | | |
| --- | --- | --- | --- | --- | --- | --- |
|  |  |  | *Fine motor concern* | *Gross motor concern* | *Communication concern* | *Social skills concern* |
| **Unadjusted analysis** | <3^rd^ | 0.78 (0.67 to 0.89) | 2.66 (2.44 to 2.86) | 2.20 (1.95 to 2.42) | 0.74 (0.62 to 0.84) | 1.06 (0.87 to 1.24) |
|  | 3^rd^ – 9^th^ | 0.75 (0.54 to 1.02) | 2.54 (2.04 to 3.07) | 2.07 (1.47 to 2.63) | 0.75 (0.54 to 1.02) | 1.68 (1.24 to 2.13) |
|  | 10^th^ – 24^th^ | 0.15 (-0.15 to 0.58) | 1.81 (1.03 to 2.72) | 0.98 (0.00 to 1.84) | 0.00 (-0.30 to 0.43) | 0.87 (0.15 to 1.53) |
|  | 75^th^ – 89^th^ | 0.00 (-0.25 to 0.36) | -1.14 (-2.11 to -0.43) | -1.40 (-2.26 to -0.54) | 0.12 (-0.13 to 0.48) | -0.60 (-1.27 to 0.11) |
|  | 90^th^ – 96^th^ | 0.11 (-0.12 to 0.32) | -0.37 (-0.93 to 0.10) | -0.64 (-1.25 to -0.10) | 0.22 (0.00 to 0.43) | -0.11 (-0.53 to 0.26) |
|  | ≥97^th^ | 0.29 (0.13 to 0.43) | 0.12 (-0.27 to 0.47) | -0.15 (-0.59 to 0.24) | 0.35 (0.17 to 0.48) | 0.25 (-0.07 to 0.52) |
|  | | | | | | |
| **Adjusted analysis** ^¥^ | <3^rd^ | 0.78 (0.58 to 0.95) | 2.54 (2.09 to 2.92) | 2.39 (1.96 to 2.73) | 0.72 (0.50 to 0.91) | 1.00 (0.66 to 1.30) |
|  | 3^rd^ – 9^th^ | 1.02 (0.69 to 1.38) | 2.89 (1.98 to 3.75) | 2.53 (1.53 to 3.41) | 1.21 (0.75 to 1.55) | 1.86 (1.11 to 2.53) |
|  | 10^th^ – 24^th^ | 0.85 (0.29 to 1.36) | 2.94 (1.43 to 4.16) | 1.73 (0.00 to 3.10) | 0.71 (0.15 to 1.35) | 1.53 (0.45 to 2.57) |
|  | 75^th^ – 89^th^ | 0.12 (-0.38 to 0.59) | 0.40 (-0.78 to 1.50) | -0.21 (-1.54 to 1.02) | 0.24 (-0.25 to 0.81) | 0.54 (-0.35 to 1.49) |
|  | 90^th^ – 96^th^ | -0.06 (-0.36 to 0.27) | 0.10 (-0.73 to 0.82) | -0.15 (-1.03 to 0.58) | 0.00 (-0.37 to 0.33) | -0.05 (-0.67 to 0.58) |
|  | ≥97^th^ | 0.20 (-0.04 to 0.43) | 0.38 (-0.20 to 0.85) | 0.24 (-0.36 to 0.72) | 0.20 (-0.07 to 0.43) | 0.39 (-0.07 to 0.78) |

¥ - Adjustment for maternal age, BMI, parity, year of birth, gestational age at delivery, child’s sex, smoking, substance misuse in pregnancy, alcohol intake, socioeconomic status [deprivation index], ethnicity, diabetes, pre-eclampsia, maternal infection during pregnancy, history of stillbirth and spontaneous abortion, and induction of labour) referent to birthweight between 25^th^ and 74^th^ centile.

Result presented as percent (95% CI)
